# Tissue-specific clustering of genetic correlations across autoimmune diseases in a nationwide sibling study

**DOI:** 10.1172/JCI205952

**Published:** 2026-07-28

**Authors:** Daniel Eriksson, Ralf Kuja-Halkola, Marie Holmqvist, Henrik Larsson, Agnieszka Butwicka, Soffia Gudbjörnsdottir, Olle Kämpe, Sophie Bensing, Jakob Skov

**Affiliations:** 1 Department of Immunology, Genetics and Pathology, Uppsala University, Uppsala, Sweden.; 2 Department of Medical Epidemiology and Biostatistics and; 3 Department of Medicine, Solna, Karolinska Institutet, Stockholm, Sweden.; 4 Department of Rheumatology, Karolinska University Hospital, Stockholm, Sweden.; 5 School of Medical Sciences, Örebro University, Örebro, Sweden.; 6 Division of Mental Health Services, Akershus University Hospital, Lørenskog, Norway.; 7 Institute of Clinical Medicine, University of Oslo, Oslo, Norway.; 8 Department of Biostatistics and Translational Medicine, Medical University of Lodz, Lodz, Poland.; 9 Department of Medical Sciences, Child and Adolescent Psychiatry, Uppsala University, Uppsala, Sweden.; 10 Department of Molecular and Clinical Medicine, University of Gothenburg, Gothenburg, Sweden.; 11 Department of Endocrinology, Karolinska University Hospital, Stockholm, Sweden.; 12 Center for Clinical Research, Region Värmland, Karlstad, Sweden.

**Keywords:** Autoimmunity, Genetics, Immunology, Autoimmune diseases, Epidemiology, Genetic risk factors

## Abstract

**BACKGROUND:**

Autoimmune diseases (ADs) often co-occur within individuals and families, indicating shared genetic risk factors. However, the composition of genetic overlap across autoimmunity is largely unknown.

**METHODS:**

This nationwide study included 6,336,615 individuals born in Sweden between 1932 and 1983, comprising 3,839,400 full sibling pairs. Based on national health registers, 22 ADs were identified from 1969 to 2013. Aggregation and coaggregation of ADs among siblings was used to estimate pairwise genetic correlations of ADs under a liability-threshold model. Network analysis and principal component analysis were used to characterize the structure of shared genetic risk across ADs.

**RESULTS:**

A total of 707,995 individuals (11.2%) were diagnosed with at least 1 AD. The studied ADs formed a network of significant genetic correlations (mean *r_g_* = 0.24, range 0.08–0.84) with clusters of more closely related ADs (*r_g_* ≥ 0.3). We found no evidence of a significant universal factor predisposing to autoimmunity.

**CONCLUSION:**

This study demonstrates that ADs share substantial cluster-specific genetic overlap that largely aligns with affected tissue types, leading to distinct groupings of connective tissue diseases, gastrointestinal disorders, and endocrinopathies, whereas diseases of the nervous system show limited genetic cohesion. This suggests that shared biological mechanisms may drive coaggregation within disease groups. Clinically, these insights highlight the importance of monitoring patients and their relatives for related autoimmune disorders.

**FUNDING:**

The Swedish Society of Medicine, Region Värmland’s County Research Council, The Swedish Research Council, The Knut and Alice Wallenberg Foundation, Regional Agreement on Medical Training and Clinical Research (ALF) between Stockholm County Council and Karolinska Institutet.

## Abstract 2

Autoimmune diseases share common origins. This study examines genetic relatedness across autoimmunity using population registers, and find that groups of disorders form separate clusters.

## Graphical abstract

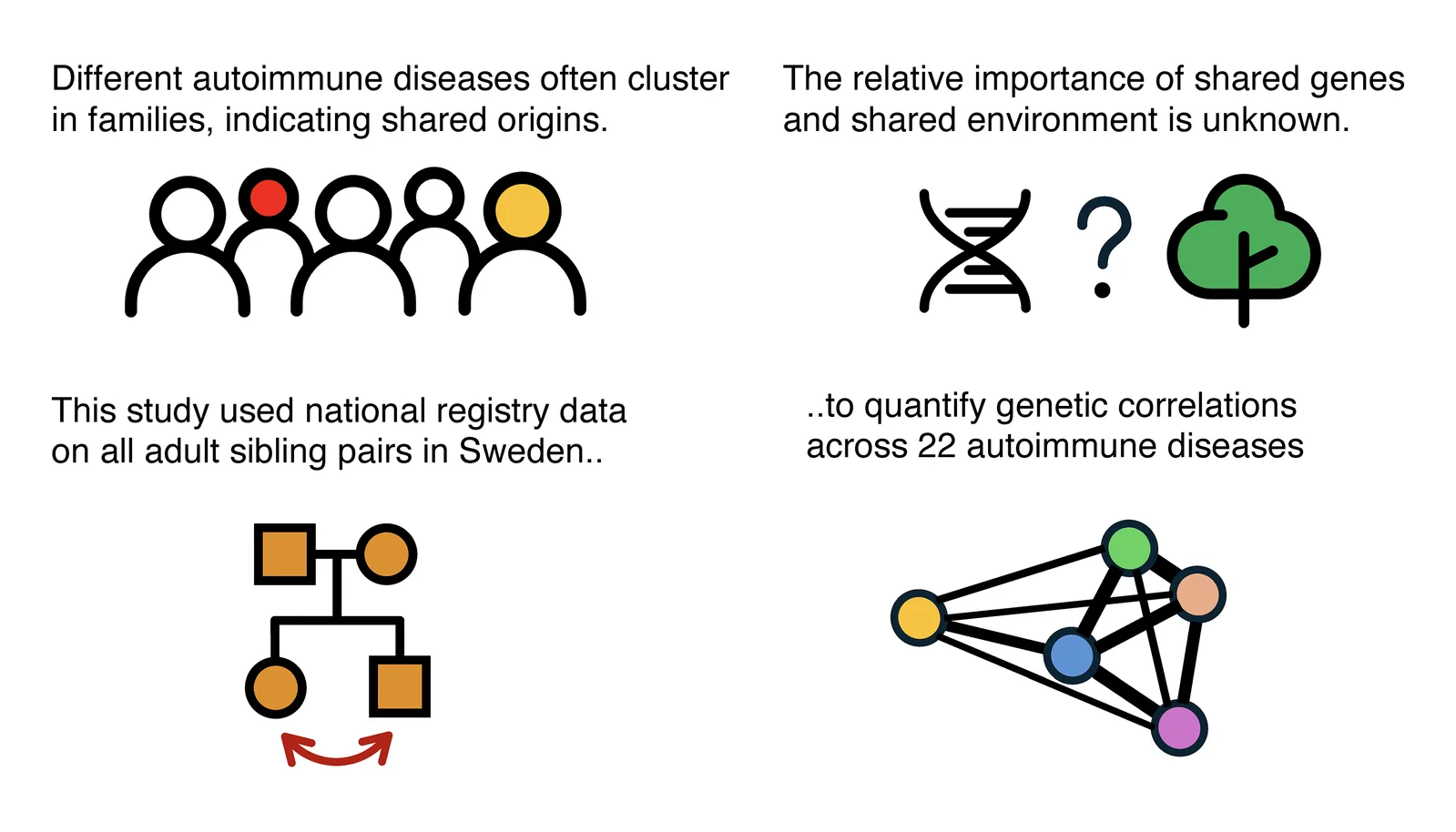

## Introduction

Autoimmune diseases (ADs) are a consequence of immune system dysfunction, characterized by a failure in self-tolerance that prompts an immune response against the body’s own tissues. Over 80 ADs have been identified, collectively affecting 5%–10% of the global population (1–3). While many ADs are rare, certain conditions, such as autoimmune thyroid disorders, rheumatoid arthritis, and psoriasis, account for a large portion of these diseases (2, 3).

ADs are generally multifactorial, influenced by a mix of genetic and environmental factors that contribute to disease susceptibility. Epidemiological studies have demonstrated that different ADs often co-occur within families, suggesting that relatives are at increased risk not only for a particular disease, but for a spectrum of autoimmune conditions (2, 4). This pattern indicates shared genetic or environmental risk factors across ADs.

A central question in AD research is whether susceptibility to autoimmunity partly arises from a common overarching genetic predisposition and to what extent subsets of ADs share genetic causes (5). ADs are often grouped based on common features, such as organ specificity, anatomic distribution, or the presence of pathogenic autoantibodies. It remains unclear, however, to what extent the similarities within these groups reflect shared genetic risk factors.

The multifactorial nature and the rarity of many ADs have long hampered large-scale studies on etiologic factors. Registry-based studies offer a broad population coverage but cannot differentiate between genetic and environmental causes without access to relatedness data. Molecular genetic studies, such as GWAS, have enabled direct identification of shared risk alleles in several ADs but are often constrained by sample size, which restricts their scope to common ADs and common risk factors (6, 7). Even the largest biobanks are too small for studying the less common ADs and furthermore susceptible to selection bias (8, 9). Consequently, a comprehensive picture of shared genetic risk factors for ADs remains elusive.

In this study, we utilized longitudinally collected diagnoses together with a nation-wide registry of kinship to investigate familial coaggregation and genetic correlations across multiple ADs in the Swedish population. Based on this information, we examined the extent to which genetic predisposition to autoimmunity is explained by a common factor or by multiple distinct components.

## Results

### Study population and AD prevalence.

From 1969 through 2013, a total of 805,745 AD diagnoses were recorded in 707,995 affected individuals, corresponding to 11.2% of the total cohort of 6,336,615 individuals. Hashimoto’s thyroiditis was the most prevalent disorder, whereas Addison’s disease was the least common, affecting 5% and 0.02% of the cohort, respectively (Table 1). The overall prevalence of ADs was higher in women (15.3%) than in men (7.2%) and increased with age, ranging from 7.5% in the youngest cohort (born 1972–1983) to 15.9% in the cohort born 1932–1941 (Supplemental Table 1; supplemental material available online with this article; https://doi.org/10.1172/JCI205952DS1). In all, 83,720 (1.3%) individuals harbored more than 1 AD (Supplemental Table 2). Within the studied cohort, 4,026,697 individuals had at least 1 full sibling, allowing us to study 3,839,400 unique sibling pairs nested in 1,574,407 nuclear families.

### Co-occurrence of ADs within individuals.

Out of 231 possible binary AD combinations, 228 were recorded at least once within an individual (Figure 1A and Supplemental Table 3). Well-established comorbidities were confirmed, e.g., psoriatic arthritis was predominantly observed among patients with psoriasis, prevalent in 0.45% of the overall cohort and in 15.1% of the subgroup diagnosed with psoriasis. Conversely, among patients diagnosed with psoriatic arthritis, 47.8% were concurrently diagnosed with psoriasis (Figure 1B). A 2.9% comorbidity rate of dermatitis herpetiformis was noted among patients with celiac disease, with a 33.9% prevalence of celiac disease in patients diagnosed with dermatitis herpetiformis (Figure 1C). Primary biliary cholangitis was observed in 4.0% of patients with autoimmune hepatitis, while 20.2% of individuals with primary biliary cholangitis also carried a diagnosis of autoimmune hepatitis (Figure 1D). No intra-individual overlap was identified for combinations of the rarest disorders, for instance, Addison’s disease with idiopathic inflammatory myopathy (Figure 1E).

ORs adjusted for age and sex (aORs) revealed patterns of disease aggregation, as depicted in Figure 2A. Crude ORs and aORs are detailed in Supplemental Table 4, A and B. Notable clusters with higher-than-expected aORs were observed among connective tissue diseases, among ADs affecting the liver, and between autoimmune endocrine disorders. Several pairs of disorders showed an aOR above 50, e.g., psoriasis with psoriatic arthritis (aOR = 66.8) and systemic lupus erythematosus with idiopathic inflammatory myopathy (aOR = 52.2). The strongest association was seen for celiac disease with dermatitis herpetiformis (aOR = 141.2). ORs for appendicitis were consistently low in this context, ranging from 0.9 to 1.8.

### Familial aggregation and coaggregation in siblings.

All disease combinations were observed across sibling pairs, except for the ultrarare concurrence of Addison’s disease with granulomatous polyangiitis.

We observed aggregation for all studied diseases (Supplemental Table 5, A and B). Specifically, aggregation aORs, i.e., the aOR of observing the same disease in a sibling, was lowest at 2.1, for primary sclerosing cholangitis. In contrast, the highest aORs were observed for systemic lupus erythematosus (aOR = 11.3), ankylosing spondylitis (aOR = 14.9), primary biliary cholangitis (aOR = 18.6), and Addison’s disease (aOR = 98.6), as depicted on the diagonal of Figure 2B.

Expanding our analysis to coaggregation, we confirmed that individuals are at heightened risk not only for the same AD observed in siblings, but for a broader spectrum of autoimmune conditions. The observed aORs varied, as depicted by cells outside of the diagonal in Figure 2B and detailed in Supplemental Table 5B. Notably, we observed the strongest associations between Addison’s disease and type 1 diabetes (aOR = 3.9), systemic lupus erythematosus and primary Sjögren’s syndrome (aOR = 3.6), primary biliary cholangitis and autoimmune hepatitis (aOR = 3.3), and celiac disease with dermatitis herpetiformis (aOR = 4.7). Adjusted ORs substantially below 1 were rare, observed only in conjunction with uncommon disorders and not statistically significant. Overall, patterns of coaggregation in siblings were highly resemblant of patterns of co-occurrence in individuals.

### Genetic correlations across ADs.

Genetic correlations (*r_g_*) were estimated for 250 disease pairs, crude and adjusted for sex and age. Three disease pairs were excluded due to optimization problems: Addison’s disease and idiopathic inflammatory myopathy, Addison’s disease and granulomatosis with polyangiitis, and primary biliary cholangitis and granulomatosis with polyangiitis (Figure 3A and Supplemental Table 6, A and B). The distribution of adjusted genetic correlations across ADs and between appendicitis and ADs, including nonsignificant estimates, is depicted in Figure 3B. For ADs, the mean correlation was *r_g_* = 0.14. By contrast, correlations between appendicitis and ADs were consistently weaker (mean *r_g_* = 0.05, range [–0.13, 0.19]) Restricting the analysis to significant genetic links (Figure 3C) amplified this difference (mean adjusted *r_g_* = 0.24 and *r_g_* = 0,02 between ADs, and between appendicitis and ADs, respectively) (Figure 3C). Among the 90 statistically significant links, the strongest adjusted correlations were noted between psoriasis and psoriatic arthritis (*r_g_* = 0.84, 95% CI [0.82, 0.87]), autoimmune hepatitis and primary biliary cholangitis (*r_g_* = 0.68, 95% CI [0.48, 0.87]), and systemic lupus erythematosus and Sjögren’s syndrome (*r_g_* = 0.61, 95% CI [0.52, 0.70]).

Several disease pairs, often involving multiple sclerosis, showed negligible genetic overlap, with CIs closely centered around 0 (95% CI [–0.1, 0.1]). Notably, type 1 diabetes and psoriasis, 2 common ADs, demonstrated marginal genetic overlap (*r_g_* = 0.026, 95% CI [–0.01, 0.06]) (Supplemental Table 7).

### Principal component analysis.

Using principal component analysis (PCA) of the genetic correlation matrix, we quantified whether the observed correlations support a common susceptibility or multiple independent patterns. The first principal component explained 21%, and the first 6 explained two-thirds of the observed variance. Overall, the PCA indicated only modest explanatory capacity from any single component, suggesting a mix of common susceptibility and independent patterns (Figure 4).

### Network analysis.

To capture the overall structure of genetic overlap among ADs, we allowed the pattern of genetic correlations to determine how the diseases grouped together in our network analysis. This data-driven approach revealed 12 distinct clusters (Figure 5A), each represented by a distinct color in the network (Figure 5, B and C). Additionally, we visualized individual links and their magnitudes in circos plots to help disentangle the densely interconnected nodes, using increasing thresholds of genetic correlation (*r_g_* = 0.10–0.45) (Figure 5, D and E, and Supplemental Figures 1 and 2). At the lowest threshold (*r_g_* ≥ 0.10), all ADs demonstrated significant links with multiple other ADs. Genetic correlations between appendicitis and ADs were consistently below this threshold (Figure 5, B and D). A threshold of *r_g_* ≥ 0.30 separated ADs into clusters of connective tissue diseases, endocrine disorders, and gastrointestinal autoimmune disorders, while diseases of the nervous system showed no propensity for internal clustering (Figure 5, C and E). Interestingly, sarcoidosis, not considered a typical AD (10), demonstrated considerable associations with Addison’s disease and dermatitis herpetiformis, as well as with multiple ADs at lower thresholds.

### External validity.

In recent years, many genetic correlations have been derived from GWAS data. To contextualize our results, we compared sibling-based genetic correlations with previously published GWAS-based estimates (7), restricted to 6 prevalent ADs. A strong positive correlation was observed (*r* = 0.84, 95% CI [0.57, 0.95]) (Supplemental Figures 3 and 4).

Specific haplotypes of the human leukocyte antigen (HLA) confer risk for ADs and explain much of the known genetic liability to autoimmunity. Supplemental Table 8 outlines known associations between HLA alleles and the ADs under study. Several genetic clusters in this study are mirrored by HLA haplotypes known to confer risk across these ADs, such as DRB1*03:01-DQA1*05:01-DQB1*02:01 for endocrine diseases, celiac disease, and dermatitis herpetiformis, and DRB1*04 for rheumatoid arthritis and granulomatosis with polyangiitis. Conversely, several of the diseases with weaker observed correlations are characterized by more specific HLA associations, such as DQB1*03:02 with myastenia gravis and DRB1*15:01-DQA1*01:02-DQB1*06:02 with multiple sclerosis.

## Discussion

In this nationwide sibling study of more than 6 million individuals, we provide a comprehensive assessment of genetic correlations across a broad range of ADs. While we find no evidence of a substantial universal genetic factor predisposing to autoimmunity, the 22 ADs examined are interconnected through a dense web of genetic links that cover the whole autoimmune panorama. When we narrow our analysis to stronger genetic links, disease clusters emerge, interlinked by individual ADs. These clusters are defined solely by their genetic correlations, but align closely with the type of tissue affected, suggesting that specific genetic risk factors are shared within groups of related disorders. Consequently, connective tissue diseases, endocrinopathies, inflammatory bowel diseases, and hepatic disorders form separate clusters. In contrast, ADs affecting the nervous system show little genetic cohesion and generally modest genetic correlations with other disorders.

In total, 11% of the observed population in this study was affected by autoimmunity, with close to 1 in 6 women diagnosed with at least 1 AD. This prevalence is considerably higher than the 5.5% reported in the Danish population by Eaton et al. (2), but consistent with more recent findings from the United Kingdom by Conrad et al., who estimated a population prevalence of 10.2% across 19 autoimmune disorders (3).

The overall magnitude and pattern of co-occurrence of ADs in this study were consistent with results from previous studies, demonstrating clustering of connective tissue diseases. However, contrary to findings from Danish registers but in line with UK data, we saw no clustering of neurologic diseases but substantial clustering of endocrine disorders (2, 3, 11). Sibling aggregation of ADs was consistent with estimates reported by Eaton et al. for most but not all diseases (2). While epidemiological studies of genetic correlations between ADs are rare, sibling-based estimates of genetic overlap between Graves’ disease, Hashimoto’s thyroiditis, and type 1 diabetes in this study align with previous results from twin studies (12, 13).

Many of the strongest genetic correlations involved well-established disease pairs, such as psoriatic arthritis and psoriasis, or dermatitis herpetiformis and celiac disease, consistent with prior findings (3, 4). Addison’s disease, type 1 diabetes, and Hashimoto’s thyroiditis, which together constitute autoimmune polyendocrine syndrome type 2 (APS2), shared substantial genetic origins (14, 15). These clusters reflect known HLA associations: APS2-related diseases with DQB1*02:01 and DQB1*03:02 (16), psoriasis and psoriatic arthritis with C*06:02 (17), and celiac disease and dermatitis herpetiformis with DQB1*02:01 and DQB1*03:02 (5, 6). The exceptionally strong genetic correlations, however, suggest additional shared genetic risk factors.

In addition to identifying well-established disease clusters, our network analysis enabled detailed comparisons of correlated diseases, for instance, celiac disease and dermatitis herpetiformis. Despite their strong genetic correlation and their shared autoreactivity to gluten, celiac disease is more than 10 times more common than dermatitis herpetiformis. Network analysis showed that dermatitis herpetiformis exhibits more extensive connections with other ADs, including sarcoidosis, Graves’ disease, and Sjögren’s syndrome (18, 19), suggesting that dermatitis herpetiformis may require additional pleiotropic genetic risk factors to develop, contributing to the lower prevalence. Similarly, while psoriatic arthritis is closely linked to psoriasis, psoriatic arthritis also correlates with rheumatoid arthritis and ankylosing spondylitis, possibly reflecting genetic risk factors mediating a susceptibility to autoimmune arthritis.

Beyond the strong genetic correlations discussed hitherto, several AD pairs also demonstrated minimal genetic correlation. For example, type 1 diabetes and psoriasis, 2 of the most prevalent ADs, had only marginal overlap in genetic risk, consistent with GWAS results (9). Furthermore, multiple sclerosis showed an exceptionally weak genetic correlation with other autoimmune disorders. This contrasts with some previous reports of significant genetic overlap between multiple sclerosis and some ADs (6, 20), but is consistent with other findings (9, 21). Graves’ disease and myasthenia gravis, despite both being antibody mediated, showed no strong genetic link, suggesting distinct underlying mechanisms.

By studying co-occurrence of a broad range of diseases in parallel, we enabled comparisons of disease relationships. For instance, while Hashimoto’s thyroiditis and Graves’ disease are often grouped together under the umbrella “autoimmune thyroid disease” (22, 23), our findings indicate that, despite genetic similarities, they are no closer related than systemic lupus erythematosus and rheumatoid arthritis, or Sjögren’s syndrome and autoimmune hepatitis, and should consequently be studied as separate entities.

Our systematic approach allowed us to study the genetic basis of rare disease combinations previously observed in disease-specific cohorts or isolated case reports. Our findings suggest that a shared genetic predisposition contributes to clustering sarcoidosis with Addison’s disease, (24, 25), and ulcerative colitis with myasthenia gravis (26). By contrast, inflammatory bowel diseases generally demonstrated low genetic overlap with endocrinopathies, suggesting that co-occurrence primarily reflects nongenetic factors or chance (27). Comparison with GWAS*-*based genetic correlations showed notable agreement, though the disease overlap was limited. Our sibling-based approach provides a broad perspective of genetic contributions to real-world AD co-occurrence. Without requiring individual-level genotype data, this approach is particularly well suited for studying the spectrum of ADs simultaneously, including rare disease combinations. In contrast, while GWAS directly identifies common genetic variants associated with diseases, its reliance on large case-control studies and biobanks limits its ability to study rare variants and disease combinations. For common ADs, our findings are consistent with GWAS results, and as biobanks expand, a broader validation of our results will be possible.

The strengths of this study include its large, population-based design and wide scope, enabling the investigation of the genetic correlation of both common and rare ADs. By focusing on sibling pairs, we mitigate biases introduced by temporal changes in diagnostic practices and case ascertainment. The relative homogeneity of Sweden’s healthcare system, coupled with stringent diagnostic criteria, enhances the robustness and generalizability of our findings. However, several limitations must also be considered. First, sibling-based models do not allow for estimation of shared environmental (C) contributions to disease liability. Nonetheless, previous studies on Nordic populations, including cross-disease correlation studies (12, 13), have demonstrated that ADs are almost exclusively governed by additive genetic (A) and unique environmental (E) components (28, 29). In addition, while the lack of modeled C could introduce a limited bias inflating heritability estimates, the potential bias on the genetic correlations, on which our study focuses, is less clear. Second, although registry data in Sweden are of high quality, variation over time led us to restrict the analysis to sibling pairs, thereby avoiding any bias from parent–offspring comparisons. The dataset used covered nearly all chapters of the International Classification of Diseases (ICD), but the code range for dermatologic diseases was incomplete. We also lacked statistical power to perform subgroup analyses by age and sex, though these factors were accounted for in the models. Finally, diagnostic misclassifications cannot be excluded, particularly for conditions with overlapping clinical features, for instance Crohn’s disease and ulcerative colitis.

In conclusion, this sibling-based study provides a comprehensive characterization of genetic correlations across ADs in a large population, revealing that ADs share substantial cluster-specific genetic overlap, rather than a single universal genetic predisposition. The alignment of genetic correlations with affected tissue types suggests that shared biological mechanisms may drive coaggregation within disease groups. Clinically, these insights highlight the importance of monitoring patients and their relatives for related autoimmune conditions and encourage a more integrated approach to diagnosis and care across specialties.

## Methods

### Sex as a biological variable

Men and women were included in all parts of the study.

### Data sources

In this population-based study, we used nationwide Swedish registers to investigate familial relationships and the occurrence of ADs through December 31, 2013. Sweden has a tax-funded healthcare system with universal access to health care.

The Swedish Total Population Register (30), containing information on vital status and migration, was used to identify the study cohort. The Multi-Generation Register (31), which holds information on biological kinships dating back to 1932, was used to identify full sibling pairs. ADs were identified using the National Patient Register, which includes diagnoses from inpatient (starting in 1969) and secondary outpatient care (starting in 2001), coded according to the ICD, versions 8–10 during the study period. The National Patient Register has been validated for a selection of ADs with positive predictive values ranging from 74% for inflammatory bowel diseases to >95% for rheumatoid arthritis (32).

To improve disease ascertainment, we used the Prescribed Drug Register (33) to collect information on dispensed drugs (starting in 2005) for treatment of ADs. Additionally, the National Diabetes Register was used to identify study participants with type 1 diabetes (34).

### Study cohort

We identified all individuals born between 1932 and 1983, restricting inclusion to those born at least 30 years before the end of the register coverage to account for the age-dependent onset of AD (3). Furthermore, we required individuals to be alive and reside in Sweden in 1978 or later with a minimum of 5 years of follow-up, accounting for migration and vital status. To reduce cohort effects arising from temporal variation in diagnostic practices and the rarity of many ADs, analyses of familial clustering were limited to full sibling pairs (i.e., siblings sharing both biological parents), regardless of sex.

### Diseases

We included 22 ADs that could be reliably identified using register data and inclusion criteria established in previous epidemiological studies (Table 1, detailed definitions in Supplemental Table 9). While most diseases meet current definitions for autoimmunity (35), some have autoinflammatory features. For simplicity, all are referred to as ADs in this work. Appendicitis, with no known autoimmune etiology, was included as a negative control.

### Statistics

#### Descriptive.

Autoimmune diagnoses were recorded for all individuals, and disease prevalences were calculated considering all persons diagnosed with individual ADs during the study period (numerator), among all individuals included in the study (denominator). Prevalence estimates were summarized by sex and birth cohorts (1932–1941, 1942–1951, 1952–1961, 1962–1971, and 1972–1983) and by cross-disorder prevalence for all disease pairs.

#### Autoimmune co-occurrence in individuals.

We fitted a standard model of disease co-occurrence, i.e., the association between ADs within individuals, using logistic regression. The odds of observing a disease in conjunction with another disease was calculated relative to the odds of observing the other disease in individuals not diagnosed with the index disease. ORs were calculated crude and adjusted for sex and age.

#### Autoimmune aggregation and co-aggregation in siblings.

To study AD aggregation, i.e., the association of 1 disease in both siblings in a pair, and coaggregation, i.e., the association of 1 disease in sibling 1 and another disease in sibling 2, all diagnostic records were investigated across full sibling pairs (hereafter referred to as siblings) and presented as unadjusted and adjusted ORs. In families with multiple siblings, all sibling pair combinations were investigated (Figure 1A). Familial aggregation and coaggregation were estimated using the drgee package in the statistical software R (version 4.4.3).

#### Quantitative genetic analysis.

Bivariate genetic correlations, crude and adjusted for sex and age, were estimated using data on siblings under a liability-threshold model, which assumes a normally distributed liability, with diseases developing above a certain threshold due to genetic/environmental factors. Based on full sibling pairs, this model allows estimation of A and E contributions but not shared C effects. Observed tetrachoric correlations, consistent with the liability-threshold assumption, were calculated across siblings and within and across disease combinations, and used to infer *r_g_* (details in Supplemental Methods). OpenMx, in statistical software R (version 4.4.3), was used for quantitative genetic modeling.

#### PCA.

A key question is to what extent a universal shared genetic predisposition underlies AD risk and to what extent diseases have distinct risk factors. To address this question, principal components were calculated across all genetic correlations and visualized using the scree plot, a line graph that displays the amount of variance explained for each principal component, arranged in descending order. Missing genetic correlation estimates were replaced by 0 in 6 of 253 instances.

### Network analysis

To capture the complexity of genetic overlap and to identify shared genetic risk within specific disease clusters, we conducted network analyses focusing on statistically significant genetic correlations. We performed unsupervised hierarchical clustering on pairwise correlations, using the hclust function in R (version 4.3.2). The resulting dendrogram was cut at 0.77 (arbitrary cutoff) to form 12 branches (clusters), each of which was assigned a distinct color for all its nodes (diseases), thus giving identical color to genetically linked diseases. Weighted networks were visualized using igraph 2.0.2 in undirected mode. Graphs were examined for increasing thresholds of genetic correlations from 0.1 to 0.35.

### Multiple testing

To account for multiple testing for the estimated *r_g_*, the significance threshold was adjusted using the Benjamini-Yekutieli method with a false discovery rate of α = 0.05, yielding a *P* value threshold of 0.0042 (2).

### Study approval

The study was approved by the Regional Ethical Review Board in Stockholm (Dnr 2013/862-31/5) and the Swedish Ethical Review Authority (Dnr 2021-05693-02). Informed consent was waived.

### Data availability

This study is based on Swedish National Patient Register data obtained from Statistics Sweden and the Swedish National Board of Health and Welfare. Values for all data used in figures and graphs are presented either in the supplemental materials or in the Supporting Data Values file. According to country-specific regulations and laws, the authors are not allowed to share primary data with third parties.

## Author contributions

OK, SB, and JS conceived the study. DE, RKH, and JS designed the study, analyzed the data, and drafted the manuscript. RKH, HL, and SG acquired the data. DE and RKH performed statistical analyses. MH and AB, along with all authors, contributed to the critical review and interpretation of the data, the manuscript, and intellectual content.

## Conflict of interest

HL reports grants from Takeda; lecture honoraria from Takeda, Medici, and Neuraxpharm Sweden AB; and honoraria for advisory board participation from Takeda and Neuraxpharm Sweden AB. AB reports lecture honoraria from Greenlight Consulting. OK owns stock in Navinci Diagnostics AB. MH serves as an unpaid member of the Medical Advisory Board of the Myositis Association.

## Funding support

- The Swedish Society of Medicine (to JS).
- Region Värmland’s County Research Council (to JS).
- Regional Agreement on Medical Training and Clinical Research (ALF) between Stockholm County Council and Karolinska Institutet (to SB).
- The Swedish Research Council (to OK).
- The Knut and Alice Wallenberg Foundation (to OK).

## Supplementary Material

Supplemental data

ICMJE disclosure forms

Supporting data values

## Figures and Tables

**Figure 1:**
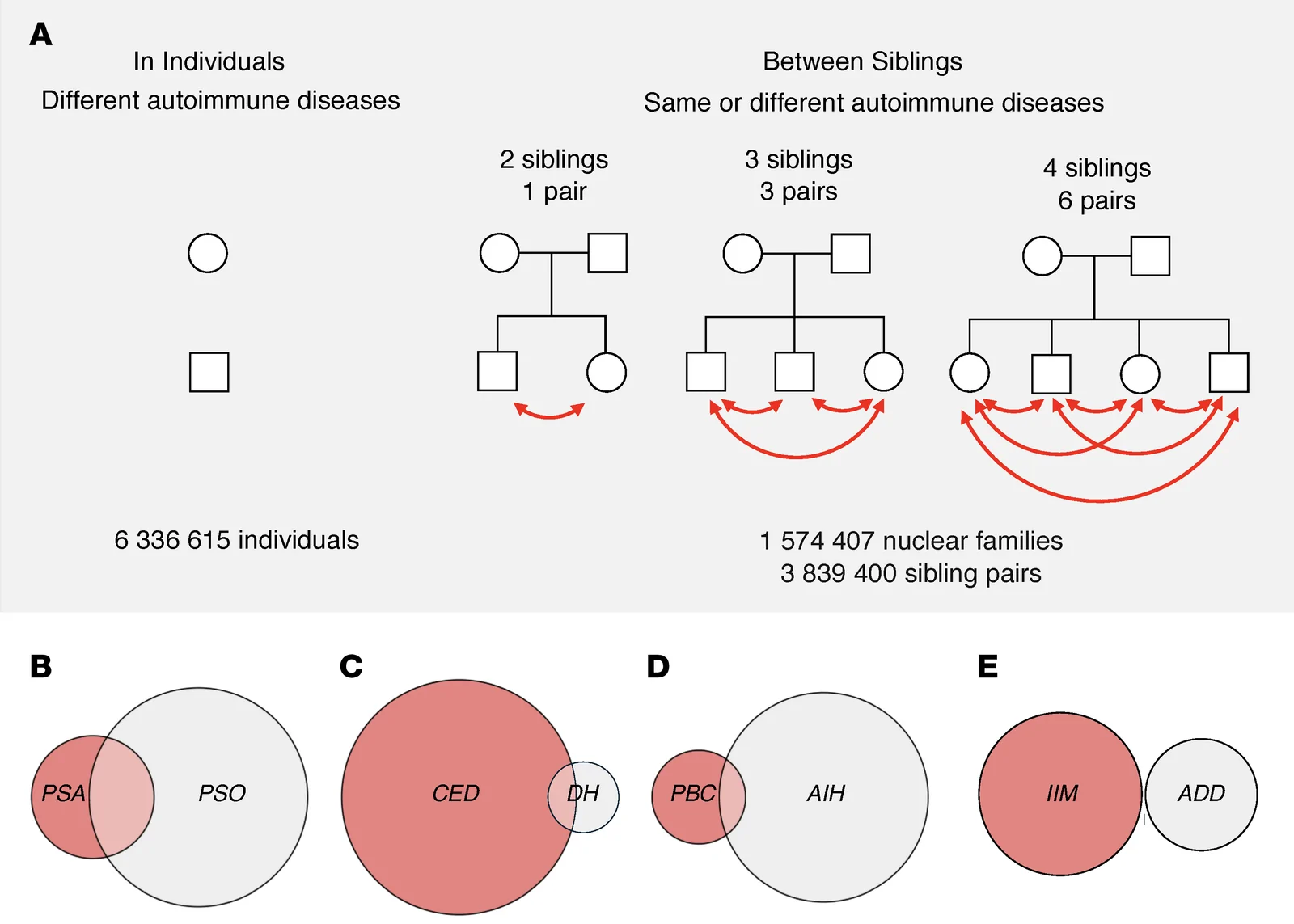
Study overview. (**A**–**E**) Number of participants and sibling pairs (**A**) and example of co-occurrence between psoriatic arthritis (PSA) and psoriasis (PSO) (**B**), celiac disease (CED) and dermatitis herpetiformis (DH) (**C**), primary biliary cholangitis (PBC) and autoimmune hepatitis (AIH) (**D**), and idiopathic inflammatory myopathy (IIM) and Addison’s disease (ADD) (**E**).

**Figure 2:**
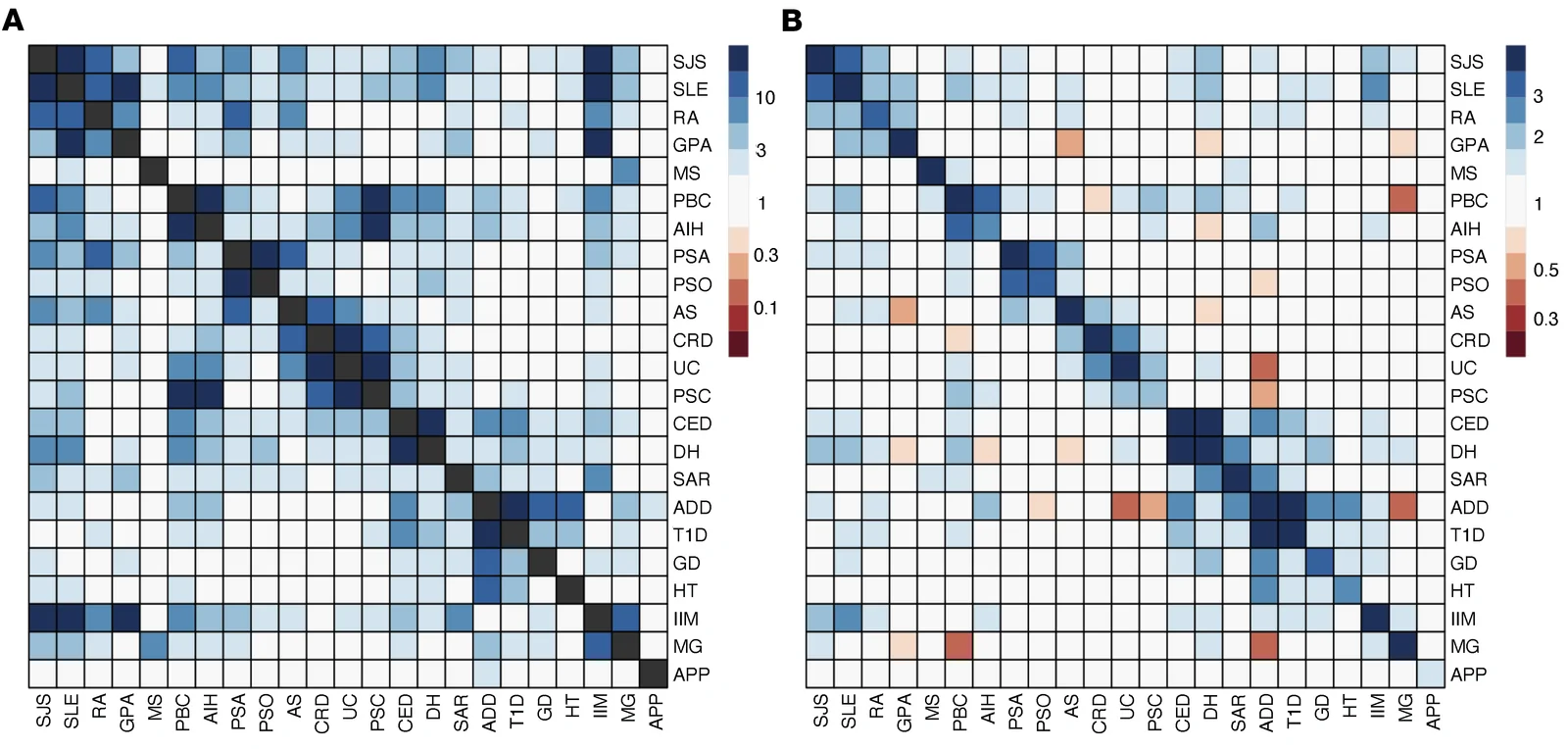
ORs for autoimmune comorbidity. (**A** and **B**) ORs for autoimmune comorbidity in individuals (**A**) and between siblings (**B**). Gradient colors indicate the magnitude of the ORs, with white representing no association (OR close to 1). ADs are ordered by hierarchical clustering based on the ORs. Abbreviations are defined in Table 1.

**Figure 3:**
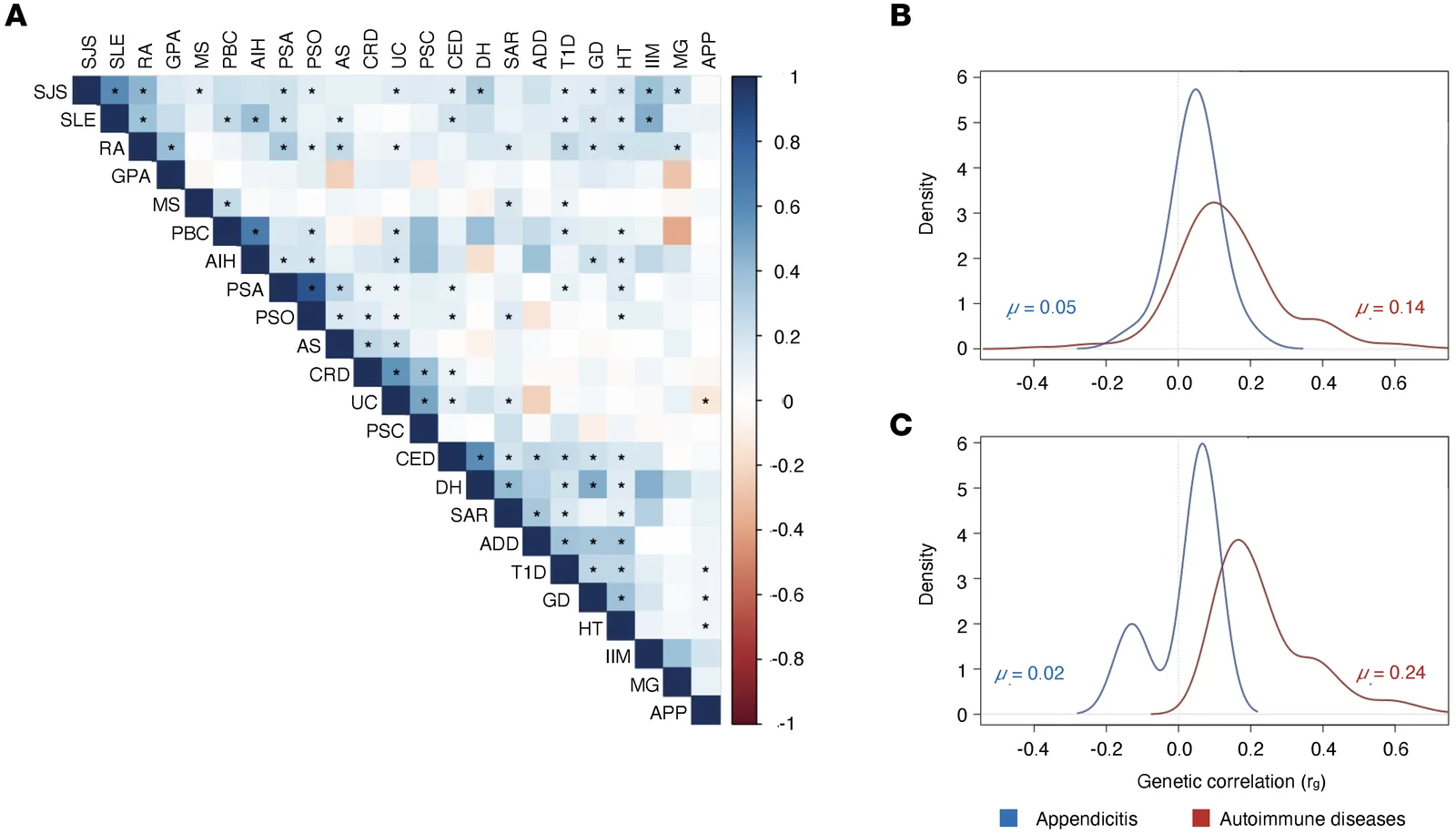
Genetic correlations between ADs. (**A**) Pairwise genetic correlation matrix. The color indicates the strength of the genetic correlations (*r_g_*), and asterisks denote statistical significance. ADs are ordered by hierarchical clustering based on the genetic correlations. (**B** and **C**) Density plots of all (**B**) and statistically significant (**C**) pairwise genetic correlations between ADs (red) and between appendicitis and ADs (blue). Abbreviations are defined in Table 1.

**Figure 4:**
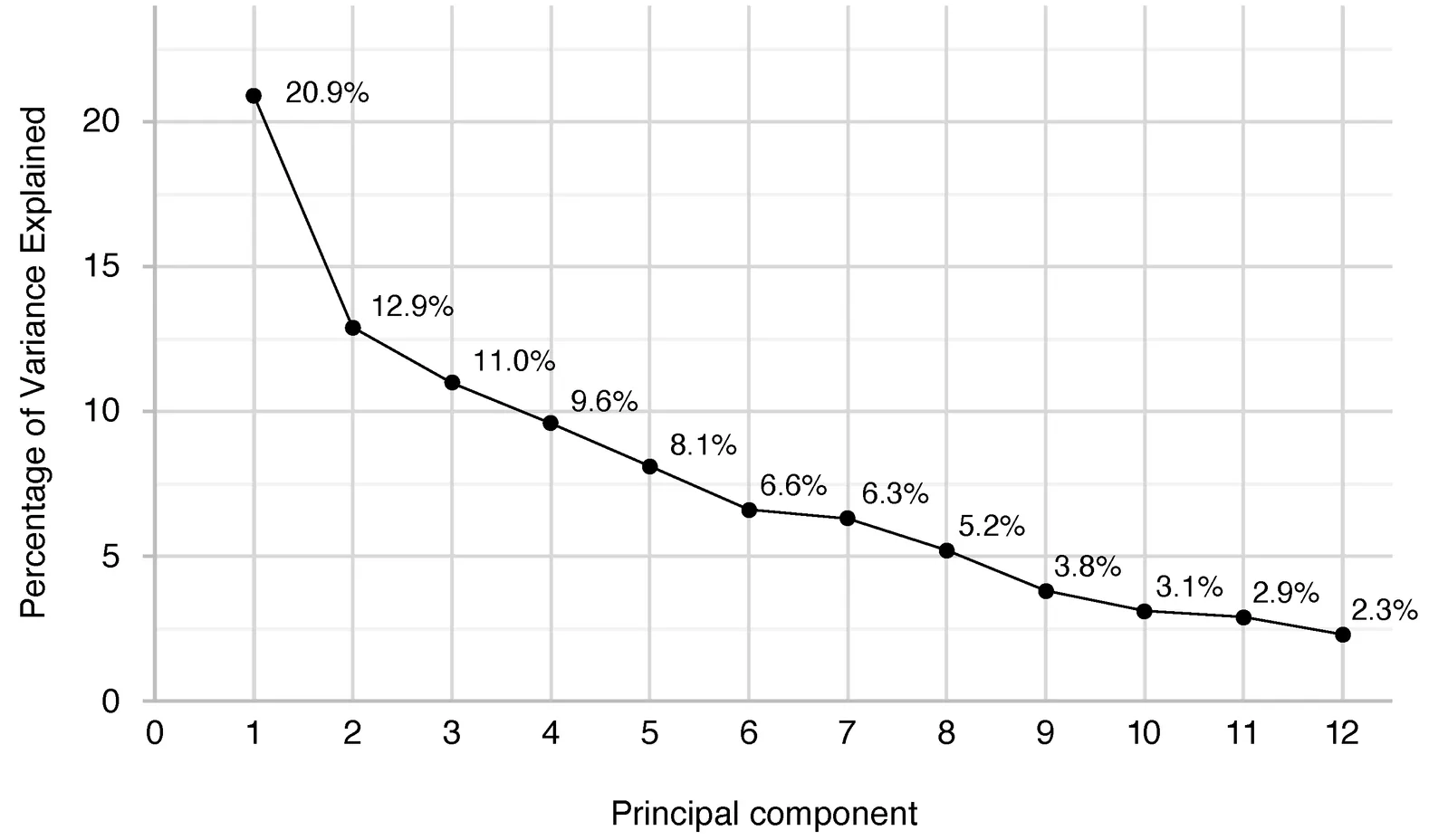
PCA. Scree plot displaying the proportion of variance explained by each principal component, based on statistically significant genetic correlations (*r_g_* ≥ 0.1). The plot illustrates the relative contribution of each component to the overall genetic structure of AD genetic correlations.

**Figure 5:**
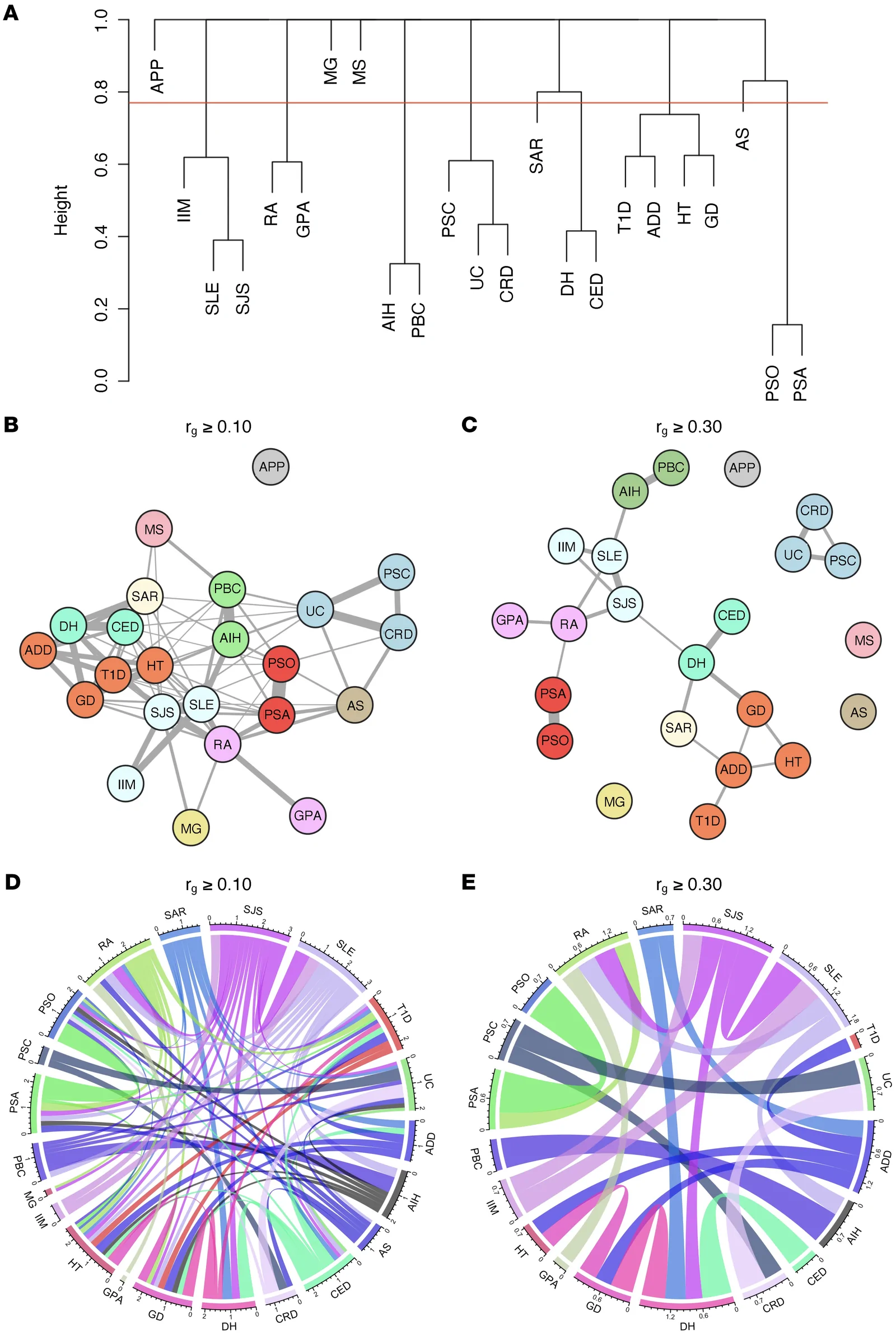
Hierarchal clustering and network analysis. Complete hierarchical clustering of statistically significant genetic correlations using the hclust function in R (version 4.3.2). (**A**) The resulting tree was cut at 0.77 to form 12 groups. (**B**–**E**) Networks (**B** and **C**) and circos plots (**D** and **E**) of genetic correlations across ADs. Abbreviations are defined in Table 1.

**Table 1:**
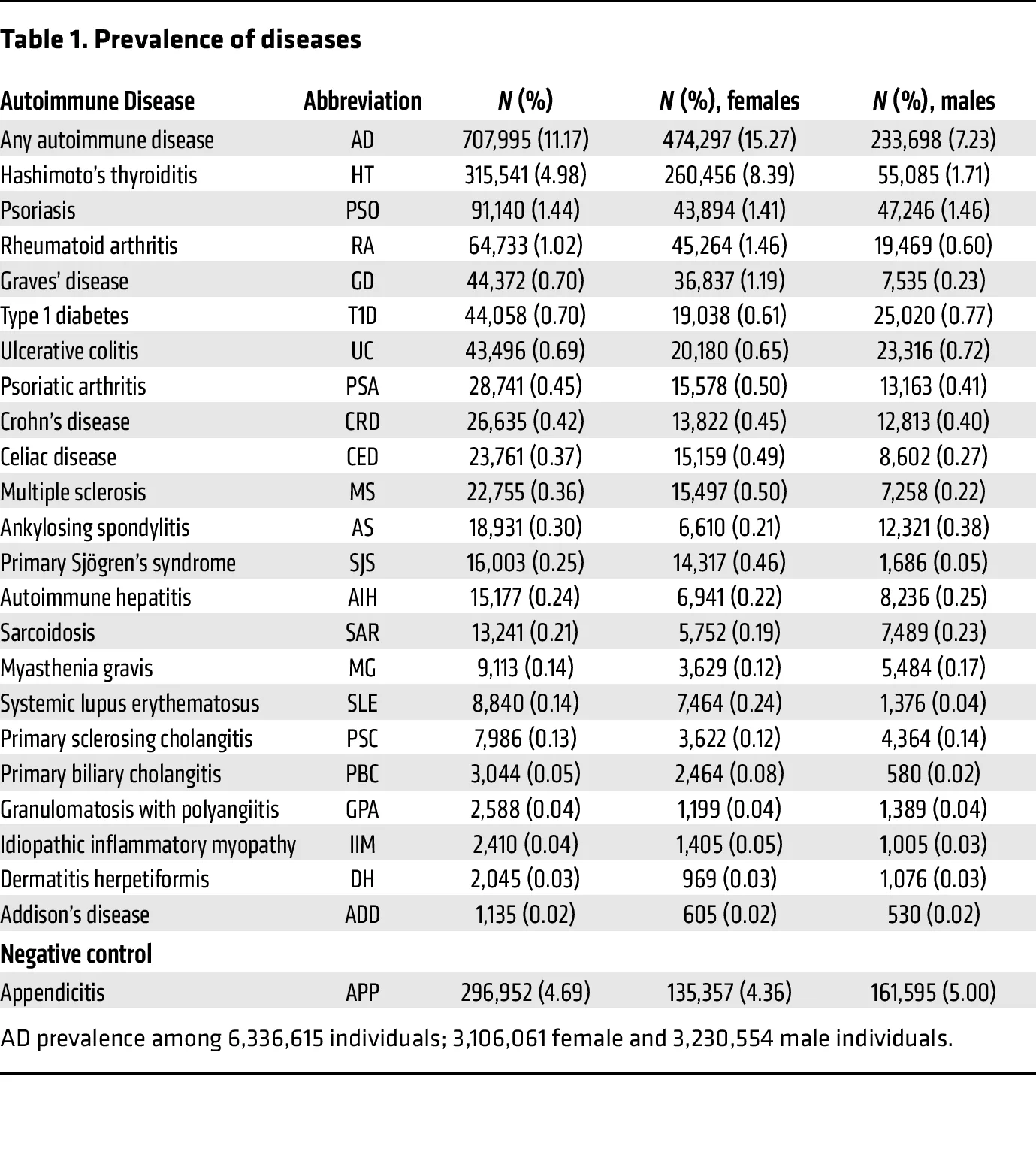
Prevalence of diseases

## Version 1 08/03/2026

In-Press Preview

## Version 2 10/01/2026

Electronic publication

## References

[1] Hayter SM, Cook MC (2012). Updated assessment of the prevalence, spectrum and case definition of autoimmune disease. Autoimmun Rev.

[2] Eaton WW (2007). Epidemiology of autoimmune diseases in Denmark. J Autoimmun.

[3] Conrad N (2023). Incidence, prevalence, and co-occurrence of autoimmune disorders over time and by age, sex, and socioeconomic status: a population-based cohort study of 22 million individuals in the UK. Lancet.

[4] Boelaert K (2010). Prevalence and relative risk of other autoimmune diseases in subjects with autoimmune thyroid disease. Am J Med.

[5] Wang L (2015). Human autoimmune diseases: a comprehensive update. J Intern Med.

[6] Lincoln MR (2024). Genetic mapping across autoimmune diseases reveals shared associations and mechanisms. Nat Genet.

[7] Demela P (2023). Cross-disorder genetic analysis of immune diseases reveals distinct gene associations that converge on common pathways. Nat Commun.

[8] Wang Y (2023). Identification of the shared genetic architecture underlying seven autoimmune diseases with GWAS summary statistics. Front Immunol.

[9] Thompson DJ (2024). A systematic evaluation of the performance and properties of the UK biobank polygenic risk score (PRS) release. PLoS One.

[10] Miedema J (2024). The immunopathogenesis of sarcoidosis. J Autoimmun.

[11] Eaton WW (2020). Comorbidity of autoimmune diseases: a visual presentation. Autoimmun Rev.

[12] Skov J (2020). Limited genetic overlap between overt Hashimoto’s thyroiditis and Graves’ disease in twins: a population-based study. J Clin Endocrinol Metab.

[13] Skov J (2022). Shared etiology of type 1 diabetes and Hashimoto’s thyroiditis: a population-based twin study. Eur J Endocrinol.

[14] Dalin F (2017). Clinical and immunological characteristics of autoimmune Addison’s disease: a nationwide Swedish multicenter study. J Clin Endocrinol Metab.

[15] Husebye ES (2018). Autoimmune polyendocrine syndromes. N Engl J Med.

[16] Eriksson D (2021). GWAS for autoimmune Addison’s disease identifies multiple risk loci and highlights AIRE in disease susceptibility. Nat Commun.

[17] Genetic Analysis of Psoriasis C (2010). A genome-wide association study identifies new psoriasis susceptibility loci and an interaction between HLA-C and ERAP1. Nat Genet.

[18] Reunala T, Collin P (1997). Diseases associated with dermatitis herpetiformis. Br J Dermatol.

[19] Kaplan RP, Callen JP (1991). Dermatitis herpetiformis: autoimmune disease associations. Clin Dermatol.

[20] Liu Q (2024). Shared aetiology underlying multiple sclerosis and other immune mediated inflammatory diseases: Swedish familial co-aggregation and large-scale genetic correlation analyses. J Autoimmun.

[21] Conrad N (2022). Autoimmune diseases and cardiovascular risk: a population-based study on 19 autoimmune diseases and 12 cardiovascular diseases in 22 million individuals in the UK. Lancet.

[22] Cooper JD (2012). Seven newly identified loci for autoimmune thyroid disease. Hum Mol Genet.

[23] Effraimidis G, Wiersinga WM (2014). Mechanisms in endocrinology: autoimmune thyroid disease: old and new players. Eur J Endocrinol.

[24] Takahashi K (2016). Sarcoidosis presenting Addison’s disease. Intern Med.

[25] Thomsen H (2020). Familial associations for Addison’s disease and between Addison’s disease and other autoimmune diseases. Endocr Connect.

[26] Ferro JM, Oliveira Santos M (2021). Neurology of inflammatory bowel disease. J Neurol Sci.

[27] Burisch J (2019). Incidence of immune-mediated inflammatory diseases among patients with inflammatory bowel diseases in Denmark. Clin Gastroenterol Hepatol.

[28] Bogdanos DP (2012). Twin studies in autoimmune disease: genetics, gender and environment. J Autoimmun.

[29] Generali E (2017). Lessons learned from twins in autoimmune and chronic inflammatory diseases. J Autoimmun.

[30] Ludvigsson JF (2016). Registers of the Swedish total population and their use in medical research. Eur J Epidemiol.

[31] Ekbom A (2011). The Swedish multi-generation register. Methods Mol Biol.

[32] Ludvigsson JF (2011). External review and validation of the Swedish national inpatient register. BMC Public Health.

[33] Wettermark B (2007). The new Swedish prescribed drug register--opportunities for pharmacoepidemiological research and experience from the first six months. Pharmacoepidemiol Drug Saf.

[34] Eeg-Olofsson K (2010). Glycemic control and cardiovascular disease in 7,454 patients with type 1 diabetes: an observational study from the Swedish national diabetes register (NDR). Diabetes Care.

[35] Rose NR, Bona C (1993). Defining criteria for autoimmune diseases (Witebsky’s postulates revisited). Immunol Today.

