# Supplementary material for: Tissue-specific clustering of genetic correlations across autoimmune diseases in a nationwide sibling study
Source: J Clin Invest. 2026 Jul 28;136(19):e205952. doi: 10.1172/JCI205952 (PMC13626829; doi:10.1172/JCI205952)
Supplement: Supplementary file 1 [file jci-136-205952-s038.pdf]

SUPPLEMENTARY INFORMATION FOR

# Tissue-specific clustering of genetic correlations across autoimmune diseases in a nationwide sibling study

## Table of Contents

*Supplemental Table S1. Disease prevalence by birth cohort.* ........................................ 2

*Supplemental Table S2. Number of autoimmune diseases per individual.* ........................................ 3

*Supplemental Table S3. Cross-disorder prevalences of autoimmune diseases.* ........................................ 4

*Supplemental Table S4A. Unadjusted odds ratios for co-occurrence of autoimmune diseases in individuals.* ........................................ 5

*Supplemental Table S4B. Adjusted odds ratios for co-occurrence of autoimmune diseases in individuals* ........................................ 6

*Supplemental Table S5A. Unadjusted odds ratios for aggregation and coaggregation across full siblings* ........................................ 7

*Supplemental Table S5B. Adjusted odds ratios for aggregation and coaggregation across full siblings.* ........................................ 8

*Supplemental Table S6A. Unadjusted genetic correlations for combinations of autoimmune diseases.* ........................................ 9

*Supplemental Table S6B. Adjusted genetic correlations for combinations of autoimmune diseases.* ........................................ 10

*Supplemental Table S7. Disease combinations with adjusted genetic correlation close to zero.* ........................................ 11

*Supplemental table S8. Overview of HLA risk alleles associated with selected autoimmune diseases* ........................................ 12

*Supplemental Table S9. List of included autoimmune diseases with register-based criteria for inclusion.* ........................................ 13

*Supplemental Figure S1. Network analyses of adjusted genetic correlations across autoimmune diseases* ........................................ 18

*Supplemental Figure S2. Circos plots of genetic correlations* ........................................ 19

*Supplemental Figure S3. Genetic correlations vs GWAS based estimates* ........................................ 20

*Supplemental Figure S4. Correlation between epidemiological genetic correlations and GWAS based estimates* ...... 21

*Supplemental Methods* ........................................ 22

**Supplemental Table S1.** Disease prevalence by birth cohort.

Number individuals (percent).

| | Birth cohorts | | | | | |
|---|---|---|---|---|---|---|
| **Disease, n (%)** | **All** n=6,336,615 | **1932-1941** n=951,384 | **1942-1951** n=1,333,452 | **1952-1961** n=1,245,215 | **1962-1971** n=1,364,138 | **1972-1983** n=1,442,426 |
| **Any autoimmune** | 707,995 (11.17) | 151,581 (15.93%) | 187,396 (14.05%) | 141,310 (11.35%) | 119,842 (8.79%) | 107,866 (7.48%) |
| Hashimoto's thyroiditis (HT) | 315,541 (4.98) | 74,866 (7.87) | 83,306 (6.25) | 60,986 (4.90) | 49,527 (3.63) | 46,856 (3.25) |
| Psoriasis (PSO) | 91,140 (1.44) | 16,718 (1.76) | 25,360 (1.90) | 18,853 (1.51) | 15,893 (1.17) | 14,316 (0.99) |
| Rheumatoid arthritis (RA) | 64,733 (1.02) | 20,895 (2.20) | 21,075 (1.58) | 11,434 (0.92) | 7,239 (0.53) | 4,090 (0.28) |
| Graves' disease (GD) | 44,372 (0.70) | 8,371 (0.88) | 11,454 (0.86) | 9,634 (0.77) | 8,641 (0.63) | 6,272 (0.43) |
| Type-1 diabetes (T1D) | 44,058 (0.70) | 5,415 (0.57) | 9,669 (0.73) | 9,314 (0.75) | 9,960 (0.73) | 9,700 (0.67) |
| Ulcerative colitis (UC) | 43,496 (0.69) | 5,837 (0.61) | 10,068 (0.76) | 9,366 (0.75) | 9,060 (0.66) | 9,165 (0.64) |
| Psoriatic arthritis (PSA) | 28,741 (0.45) | 4,272 (0.45) | 8,253 (0.62) | 7,174 (0.58) | 5,729 (0.42) | 3,313 (0.23) |
| Crohn's disease (CRD) | 26,635 (0.42) | 3,410 (0.36) | 6,397 (0.48) | 5,605 (0.45) | 5,618 (0.41) | 5,605 (0.39) |
| Celiac disease (CED) | 23,761 (0.37) | 3,814 (0.40) | 4,685 (0.35) | 3,499 (0.28) | 4,108 (0.30) | 7,655 (0.53) |
| Multiple sclerosis (MS) | 22,755 (0.36) | 3,499 (0.37) | 5,834 (0.44) | 5,179 (0.42) | 4,676 (0.34) | 3,567 (0.25) |
| Ankylosing spondylitis (AS) | 18,931 (0.30) | 3,187 (0.33) | 5,184 (0.39) | 4,295 (0.34) | 3,651 (0.27) | 2,614 (0.18) |
| Primary Sjögren's syndrome (SJS) | 16,003 (0.25) | 4,682 (0.49) | 5,529 (0.41) | 3,065 (0.25) | 1,813 (0.13) | 914 (0.06) |
| Autoimmune hepatitis (AIH) | 15,177 (0.24) | 4,664 (0.49) | 4,902 (0.37) | 3,303 (0.27) | 1,486 (0.11) | 822 (0.06) |
| Sarcoidosis (SAR) | 13,241 (0.21) | 2,157 (0.23) | 3,186 (0.24) | 2,854 (0.23) | 3,140 (0.23) | 1,904 (0.13) |
| Myasthenia gravis (MG) | 9,113 (0.14) | 2,912 (0.31) | 2,798 (0.21) | 1,744 (0.14) | 1,052 (0.08) | 607 (0.04) |
| Systemic lupus erythematosus (SLE) | 8,840 (0.14) | 1,866 (0.20) | 2,431 (0.18) | 1,743 (0.14) | 1,596 (0.12) | 1,204 (0.08) |
| Primary sclerosing cholangitis (PSC) | 7,986 (0.13) | 3,138 (0.33) | 2,392 (0.18) | 1,086 (0.09) | 760 (0.06) | 610 (0.04) |
| Primary biliary cholangitis (PBC) | 3,044 (0.05) | 1,044 (0.11) | 1,126 (0.08) | 507 (0.04) | 245 (0.02) | 122 (0.01) |
| Granulomatosis with polyangiitis (GPA) | 2,588 (0.04) | 855 (0.09) | 825 (0.06) | 418 (0.03) | 308 (0.02) | 182 (0.01) |
| Idiopathic inflammatory myopathy (IIM) | 2,410 (0.04) | 763 (0.08) | 739 (0.06) | 443 (0.04) | 290 (0.02) | 175 (0.01) |
| Dermatitis herpetiformis (DH) | 2,045 (0.03) | 641 (0.07) | 621 (0.05) | 335 (0.03) | 247 (0.02) | 201 (0.01) |
| Addison's disease (ADD) | 1,135 (0.02) | 156 (0.02) | 267 (0.02) | 249 (0.02) | 272 (0.02) | 191 (0.01) |
| Appendicitis (APP) | 296,952 (4.69) | 25,614 (2.69) | 48,796 (3.66) | 61,049 (4.90) | 81,665 (5.99) | 79,828 (5.53) |

**Supplemental Table S2.** Number of autoimmune diseases per individual.

| | All | Women | Men | 1932-1941 | 1942-1951 | 1952-1961 | 1962-1971 | 1972-1983 |
|---|---|---|---|---|---|---|---|---|
| No autoimmune disorder | 5,628,620 | 2,631,764 | 2,996,856 | 799,803 | 1,146,056 | 1,103,905 | 1,244,296 | 1,334,560 |
| At least one autoimmune disorder | 707,995 (100.00%) | 474,297 (100.00%) | 233,698 (100.00%) | 151,581 (100.00%) | 187,396 (100.00%) | 141,310 (100.00%) | 119,842 (100.00%) | 107,866 (100.00%) |
| 1 | 624,275 (88.18%) | 417,755 (88.08%) | 206,520 (88.37%) | 133,095 (87.80%) | 163,207 (87.09%) | 124,414 (88.04%) | 106,410 (88.79%) | 97,149 (90.06%) |
| 2 | 71,769 (10.14%) | 48,184 (10.16%) | 23,585 (10.09%) | 15,839 (10.45%) | 20,369 (10.87%) | 14,446 (10.22%) | 11,672 (9.74%) | 9,443 (8.75%) |
| 3 | 10,186 (1.44%) | 7,072 (1.49%) | 3,114 (1.33%) | 2,268 (1.50%) | 3,221 (1.72%) | 2,085 (1.48%) | 1,533 (1.28%) | 1,079 (1.00%) |
| 4 | 1,499 (0.21%) | 1,102 (0.23%) | 397 (0.17%) | 320 (0.21%) | 515 (0.27%) | 308 (0.22%) | 188 (0.16%) | 168 (0.16%) |
| ≥5 | 266 (0.04%) | 184 (0.04%) | 82 (0.04%) | 59 (0.04%) | 84 (0.04%) | 57 (0.04%) | 39 (0.03%) | 27 (0.03%) |
| Total number individuals | 6,336,615 | 3,106,061 | 3,230,554 | 951,384 | 1,333,452 | 1,245,215 | 1,364,138 | 1,442,426 |
| Proportion with at least one autoimmune disorder | 707,995 (11.17%) | 474,297 (15.27%) | 233,698 (7.23%) | 151,581 (15.93%) | 187,396 (14.05%) | 141,310 (11.35%) | 119,842 (8.79%) | 107,866 (7.48%) |
| Proportion with two autoimmune disorders or more | 83,720 (1.32%) | 56,542 (1.82%) | 27,178 (0.84%) | 18,486 (1.94%) | 24,189 (1.81%) | 16,896 (1.36%) | 13,432 (0.98%) | 10,717 (0.74%) |

**Supplemental Table S3.** Cross-disorder prevalences of autoimmune diseases.

Percent in parenthesis are presented by rows. Cells with counts between 1 and 4 are presented as ”<5” for non-identifiability.

| | HT | GD | T1D | ADD | PSO | PSA | DH | CED | UC | CRD | AIH | PBC | PSC | MS | MYA | RA | AS | SLE | SJS | IIM | GPA | SAR | APP |
|---|---|---|---|---|---|---|---|---|---|---|---|---|---|---|---|---|---|---|---|---|---|---|---|
| HT | 315541 | 2320 (0.7%) | 6435 (2.0%) | 452 (0.1%) | 6523 (2.1%) | 2351 (0.7%) | 243 (0.1%) | 2843 (0.9%) | 2245 (0.7%) | 1336 (0.4%) | 1160 (0.4%) | 472 (0.1%) | 561 (0.2%) | 1495 (0.5%) | 665 (0.2%) | 6603 (2.1%) | 905 (0.3%) | 1151 (0.4%) | 2878 (0.9%) | 253 (0.1%) | 216 (0.1%) | 956 (0.3%) | 14780 (4.7%) |
| GD | 2320 (5.2%) | 44372 | 983 (2.2%) | 76 (0.2%) | 778 (1.8%) | 325 (0.7%) | 30 (0.1%) | 461 (1.0%) | 411 (0.9%) | 264 (0.6%) | 160 (0.4%) | 52 (0.1%) | 90 (0.2%) | 193 (0.4%) | 118 (0.3%) | 1011 (2.3%) | 123 (0.3%) | 157 (0.4%) | 353 (0.8%) | 38 (0.1%) | 55 (0.1%) | 175 (0.4%) | 2194 (4.9%) |
| T1D | 6435 (14.6%) | 983 (2.2%) | 44058 | 155 (0.4%) | 962 (2.2%) | 294 (0.7%) | 75 (0.2%) | 1006 (2.3%) | 457 (1.0%) | 172 (0.4%) | 211 (0.5%) | 43 (0.1%) | 141 (0.3%) | 175 (0.4%) | 110 (0.2%) | 929 (2.1%) | 135 (0.3%) | 77 (0.2%) | 141 (0.3%) | 27 (0.1%) | 29 (0.1%) | 209 (0.5%) | 2815 (6.4%) |
| ADD | 452 (39.8%) | 76 (6.7%) | 155 (13.7%) | 1135 | 22 (1.9%) | 8 (0.7%) | <5 | 39 (3.4%) | 8 (0.7%) | 8 (0.7%) | 9 (0.8%) | <5 | <5 | <5 | 6 (0.5%) | 18 (1.6%) | <5 | <5 | 9 (0.8%) | 0 | 0 | 11 (1.0%) | 97 (8.5%) |
| PSO | 6523 (7.2%) | 778 (0.9%) | 962 (1.1%) | 22 (0.0%) | 91140 | 13724 (15.1%) | 111 (0.1%) | 653 (0.7%) | 1151 (1.3%) | 833 (0.9%) | 461 (0.5%) | 105 (0.1%) | 193 (0.2%) | 407 (0.4%) | 206 (0.2%) | 2722 (3.0%) | 886 (1.0%) | 244 (0.3%) | 614 (0.7%) | 94 (0.1%) | 72 (0.1%) | 367 (0.4%) | 4583 (5.0%) |
| PSA | 2351 (8.2%) | 325 (1.1%) | 294 (1.0%) | 8 (0.0%) | 13724 (47.8%) | 28741 | 20 (0.1%) | 254 (0.9%) | 443 (1.5%) | 364 (1.3%) | 152 (0.5%) | 56 (0.2%) | 62 (0.2%) | 101 (0.4%) | 83 (0.3%) | 3804 (13.2%) | 973 (3.4%) | 186 (0.6%) | 501 (1.7%) | 50 (0.2%) | 41 (0.1%) | 144 (0.5%) | 1707 (5.9%) |
| DH | 243 (11.9%) | 30 (1.5%) | 75 (3.7%) | <5 | 111 (5.4%) | 20 (1.0%) | 2045 | 694 (33.9%) | 41 (2.0%) | 22 (1.1%) | 21 (1.0%) | 8 (0.4%) | 11 (0.5%) | 8 (0.4%) | 6 (0.3%) | 44 (2.2%) | 9 (0.4%) | 18 (0.9%) | 37 (1.8%) | <5 | <5 | 14 (0.7%) | 128 (6.3%) |
| CED | 2843 (12.0%) | 461 (1.9%) | 1006 (4.2%) | 39 (0.2%) | 653 (2.7%) | 254 (1.1%) | 694 (2.9%) | 23761 | 692 (2.9%) | 465 (2.0%) | 217 (0.9%) | 88 (0.4%) | 107 (0.5%) | 104 (0.4%) | 57 (0.2%) | 447 (1.9%) | 132 (0.6%) | 141 (0.6%) | 360 (1.5%) | 45 (0.2%) | 15 (0.1%) | 133 (0.6%) | 1575 (6.6%) |
| UC | 2245 (5.2%) | 411 (0.9%) | 457 (1.1%) | 8 (0.0%) | 1151 (2.6%) | 443 (1.0%) | 41 (0.1%) | 692 (1.6%) | 43496 | 5652 (13.0%) | 621 (1.4%) | 123 (0.3%) | 1297 (3.0%) | 192 (0.4%) | 83 (0.2%) | 749 (1.7%) | 771 (1.8%) | 140 (0.3%) | 245 (0.6%) | 31 (0.1%) | 45 (0.1%) | 178 (0.4%) | 1976 (4.5%) |
| CRD | 1336 (5.0%) | 264 (1.0%) | 172 (0.6%) | 8 (0.0%) | 833 (3.1%) | 364 (1.4%) | 22 (0.1%) | 465 (1.7%) | 5652 (21.2%) | 26635 | 258 (1.0%) | 41 (0.2%) | 451 (1.7%) | 132 (0.5%) | 41 (0.2%) | 478 (1.8%) | 776 (2.9%) | 85 (0.3%) | 173 (0.6%) | 11 (0.0%) | 22 (0.1%) | 95 (0.4%) | 1675 (6.3%) |
| AIH | 1160 (7.6%) | 160 (1.1%) | 211 (1.4%) | 9 (0.1%) | 461 (3.0%) | 152 (1.0%) | 21 (0.1%) | 217 (1.4%) | 621 (4.1%) | 258 (1.7%) | 15177 | 609 (4.0%) | 434 (2.9%) | 65 (0.4%) | 86 (0.6%) | 355 (2.3%) | 99 (0.7%) | 187 (1.2%) | 171 (1.1%) | 29 (0.2%) | 20 (0.1%) | 63 (0.4%) | 677 (4.5%) |
| PBC | 472 (15.5%) | 52 (1.7%) | 43 (1.4%) | <5 | 105 (3.4%) | 56 (1.8%) | 8 (0.3%) | 88 (2.9%) | 123 (4.0%) | 41 (1.3%) | 609 (20.0%) | 3044 | 151 (5.0%) | 24 (0.8%) | 14 (0.5%) | 139 (4.6%) | 17 (0.6%) | 61 (2.0%) | 242 (8.0%) | 10 (0.3%) | 0 | 20 (0.7%) | 133 (4.4%) |
| PSC | 561 (7.0%) | 90 (1.1%) | 141 (1.8%) | <5 | 193 (2.4%) | 62 (0.8%) | 11 (0.1%) | 107 (1.3%) | 1297 (16.2%) | 451 (5.6%) | 434 (5.4%) | 151 (1.9%) | 7986 | 42 (0.5%) | 27 (0.3%) | 168 (2.1%) | 50 (0.6%) | 40 (0.5%) | 62 (0.8%) | 7 (0.1%) | 12 (0.2%) | 47 (0.6%) | 410 (5.1%) |
| MS | 1495 (6.6%) | 193 (0.8%) | 175 (0.8%) | <5 | 407 (1.8%) | 101 (0.4%) | 8 (0.0%) | 104 (0.5%) | 192 (0.8%) | 132 (0.6%) | 65 (0.3%) | 24 (0.1%) | 42 (0.2%) | 22755 | 176 (0.8%) | 206 (0.9%) | 96 (0.4%) | 79 (0.3%) | 140 (0.6%) | 14 (0.1%) | 7 (0.0%) | 72 (0.3%) | 1128 (5.0%) |
| MG | 665 (7.3%) | 118 (1.3%) | 110 (1.2%) | 6 (0.1%) | 206 (2.3%) | 83 (0.9%) | 6 (0.1%) | 57 (0.6%) | 83 (0.9%) | 41 (0.4%) | 86 (0.9%) | 14 (0.2%) | 27 (0.3%) | 176 (1.9%) | 9113 | 257 (2.8%) | 47 (0.5%) | 62 (0.7%) | 84 (0.9%) | 53 (0.6%) | 7 (0.1%) | 22 (0.2%) | 468 (5.1%) |
| RA | 6603 (10.2%) | 1011 (1.6%) | 929 (1.4%) | 18 (0.0%) | 2722 (4.2%) | 3804 (5.9%) | 44 (0.1%) | 447 (0.7%) | 749 (1.2%) | 478 (0.7%) | 355 (0.5%) | 139 (0.2%) | 168 (0.3%) | 206 (0.3%) | 257 (0.4%) | 64733 | 1494 (2.3%) | 1362 (2.1%) | 3233 (5.0%) | 313 (0.5%) | 199 (0.3%) | 269 (0.4%) | 2775 (4.3%) |
| AS | 905 (4.8%) | 123 (0.6%) | 135 (0.7%) | <5 | 886 (4.7%) | 973 (5.1%) | 9 (0.0%) | 132 (0.7%) | 771 (4.1%) | 776 (4.1%) | 99 (0.5%) | 17 (0.1%) | 50 (0.3%) | 96 (0.5%) | 47 (0.2%) | 1494 (7.9%) | 18931 | 112 (0.6%) | 273 (1.4%) | 26 (0.1%) | 17 (0.1%) | 89 (0.5%) | 882 (4.7%) |
| SLE | 1151 (13.0%) | 157 (1.8%) | 77 (0.9%) | <5 | 244 (2.8%) | 186 (2.1%) | 18 (0.2%) | 141 (1.6%) | 140 (1.6%) | 85 (1.0%) | 187 (2.1%) | 61 (0.7%) | 40 (0.5%) | 79 (0.9%) | 62 (0.7%) | 1362 (15.4%) | 112 (1.3%) | 8840 | 1364 (15.4%) | 199 (2.3%) | 83 (0.9%) | 55 (0.6%) | 489 (5.5%) |
| SJS | 2878 (18.0%) | 353 (2.2%) | 141 (0.9%) | 9 (0.1%) | 614 (3.8%) | 501 (3.1%) | 37 (0.2%) | 360 (2.2%) | 245 (1.5%) | 173 (1.1%) | 171 (1.1%) | 242 (1.5%) | 62 (0.4%) | 140 (0.9%) | 84 (0.5%) | 3233 (20.2%) | 273 (1.7%) | 1364 (8.5%) | 16003 | 170 (1.1%) | 45 (0.3%) | 136 (0.8%) | 810 (5.1%) |
| IIM | 253 (10.5%) | 38 (1.6%) | 27 (1.1%) | 0 | 94 (3.9%) | 50 (2.1%) | <5 | 45 (1.9%) | 31 (1.3%) | 11 (0.5%) | 29 (1.2%) | 10 (0.4%) | 7 (0.3%) | 14 (0.6%) | 53 (2.2%) | 313 (13.0%) | 26 (1.1%) | 199 (8.3%) | 170 (7.1%) | 2410 | 28 (1.2%) | 31 (1.3%) | 104 (4.3%) |
| GPA | 216 (8.3%) | 55 (2.1%) | 29 (1.1%) | 0 | 72 (2.8%) | 41 (1.6%) | <5 | 15 (0.6%) | 45 (1.7%) | 22 (0.9%) | 20 (0.8%) | 0 | 12 (0.5%) | 7 (0.3%) | 7 (0.3%) | 199 (7.7%) | 17 (0.7%) | 83 (3.2%) | 45 (1.7%) | 28 (1.1%) | 2588 | 32 (1.2%) | 113 (4.4%) |
| SAR | 956 (7.2%) | 175 (1.3%) | 209 (1.6%) | 11 (0.1%) | 367 (2.8%) | 144 (1.1%) | 14 (0.1%) | 133 (1.0%) | 178 (1.3%) | 95 (0.7%) | 63 (0.5%) | 20 (0.2%) | 47 (0.4%) | 72 (0.5%) | 22 (0.2%) | 269 (2.0%) | 89 (0.7%) | 55 (0.4%) | 136 (1.0%) | 31 (0.2%) | 32 (0.2%) | 13241 | 823 (6.2%) |
| APP | 14780 (5.0%) | 2194 (0.7%) | 2815 (0.9%) | 97 (0.0%) | 4583 (1.5%) | 1707 (0.6%) | 128 (0.0%) | 1575 (0.5%) | 1976 (0.7%) | 1675 (0.6%) | 677 (0.2%) | 133 (0.0%) | 410 (0.1%) | 1128 (0.4%) | 468 (0.2%) | 2775 (0.9%) | 882 (0.3%) | 489 (0.2%) | 810 (0.3%) | 104 (0.0%) | 113 (0.0%) | 823 (0.3%) | 296952 |

**Supplemental Table S4A.** Unadjusted odds ratios for co-occurrence of autoimmune diseases in individuals**.**
95% confidence intervals are given within parentheses.

| | GD | T1D | ADD | PSO | PSA | DH | CED | UC | CRD | AIH | PBC | PSC | MS | MG | RA | AS | SLE | SJS | IIM | GPA | SAR | APP |
|---|---|---|---|---|---|---|---|---|---|---|---|---|---|---|---|---|---|---|---|---|---|---|
| HT | 1.0 (1.0, 1.1) | 3.4 (3.3, 3.5) | 12.9(11.4, 14.6) | 1.4 (1.4, 1.5) | 1.7 (1.6, 1.8) | 2.7 (2.3, 3.1) | 2.6 (2.5, 2.7) | 1.0 (1.0, 1.1) | 1.0 (0.9, 1.0) | 1.7 (1.6, 1.8) | 3.7 (3.3, 4.1) | 1.4 (1.3, 1.5) | 1.3 (1.3, 1.4) | 1.6 (1.4, 1.7) | 2.2 (2.1, 2.2) | 0.9 (0.9, 1.0) | 2.9 (2.7, 3.1) | 4.2 (4.0, 4.4) | 2.3 (2.0, 2.7) | 1.8 (1.5, 2.0) | 1.5 (1.4, 1.6) | 1.0 (1.0, 1.0) |
| GD | | 3.4 (3.2, 3.6) | 10.2 (8.0, 13.0) | 1.2 (1.1, 1.3) | 1.7 (1.5, 1.9) | 2.3 (1.6, 3.3) | 2.9 (2.6, 3.1) | 1.4 (1.2, 1.5) | 1.5 (1.3, 1.7) | 1.6 (1.4, 1.9) | 2.6 (2.0, 3.5) | 1.7 (1.4, 2.1) | 1.2 (1.0, 1.4) | 2.0 (1.6, 2.4) | 2.3 (2.2, 2.5) | 0.9 (0.7, 1.1) | 2.7 (2.2, 3.2) | 3.3 (3.0, 3.7) | 2.3 (1.6, 3.3) | 3.0 (2.2, 4.0) | 1.9 (1.6, 2.2) | 1.0 (1.0, 1.1) |
| T1D | | | 21.2 (17.8, 25.2) | 1.5 (1.4, 1.6) | 1.4 (1.2, 1.6) | 5.1 (4.0, 6.5) | 6.0 (5.6, 6.4) | 1.4 (1.3, 1.6) | 0.9 (0.8, 1.1) | 2.1 (1.8, 2.4) | 2.1 (1.5, 2.9) | 2.5 (2.1, 3.0) | 1.1 (0.9, 1.3) | 1.8 (1.5, 2.1) | 2.1 (2.0, 2.3) | 1.0 (0.8, 1.1) | 1.2 (0.9, 1.5) | 1.2 (1.0, 1.5) | 1.5 (1.0, 2.2) | 1.5 (1.0, 2.2) | 2.1 (1.8, 2.4) | 1.3 (1.3, 1.4) |
| ADD | | | | 1.3 (0.8, 2.0) | 1.5 (0.8, 3.0) | 2.8 (0.4, 19.6) | 8.9 (6.5, 12.3) | 1.0 (0.5, 2.0) | 1.6 (0.8, 3.3) | 3.7 (1.8, 7.7) | 3.9 (1.0, 15.8) | 1.5 (0.4, 6.1) | 1.0 (0.4, 2.6) | 3.8 (1.7, 8.6) | 1.4 (0.9, 2.4) | 0.9 (0.3, 2.7) | 2.0 (0.6, 6.2) | 2.9 (1.5, 5.9) | 0.0 (0.0, 0.0) | 0.0 (0.0, 0.0) | 3.4 (1.7, 6.8) | 1.8 (1.4, 2.2) |
| PSO | | | | | 68.9 (67.2, 70.7) | 3.6 (2.9, 4.4) | 1.8 (1.7, 2.0) | 1.8 (1.7, 1.9) | 2.1 (2.0, 2.3) | 2.1 (1.9, 2.3) | 2.5 (2.0, 3.1) | 1.7 (1.5, 2.0) | 1.2 (1.1, 1.3) | 1.5 (1.3, 1.7) | 3.0 (2.9, 3.1) | 3.2 (3.0, 3.5) | 2.0 (1.7, 2.2) | 2.7 (2.5, 3.0) | 2.6 (2.1, 3.3) | 1.8 (1.4, 2.4) | 1.9 (1.7, 2.1) | 1.0 (1.0, 1.1) |
| PSA | | | | | | 2.1 (1.4, 3.4) | 2.2 (2.0, 2.5) | 2.1 (1.9, 2.3) | 2.9 (2.6, 3.2) | 2.3 (1.9, 2.7) | 4.0 (3.0, 5.3) | 1.9 (1.4, 2.4) | 0.9 (0.8, 1.1) | 2.0 (1.6, 2.5) | 14.9(14.4, 15.5) | 11.4(10.6, 12.2) | 4.6 (4.0, 5.4) | 7.0 (6.3, 7.6) | 4.6 (3.5, 6.2) | 3.6 (2.7, 5.0) | 2.3 (1.9, 2.7) | 1.2 (1.2, 1.3) |
| DH | | | | | | | 129.3 (117.2, 142.7) | 2.6 (1.9, 3.7) | 2.4 (1.6, 3.8) | 4.9 (3.2, 7.7) | 8.2 (3.9, 17.2) | 4.5 (2.4, 8.4) | 1.0 (0.5, 2.1) | 1.9 (0.8, 4.6) | 2.2 (1.6, 3.0) | 1.6 (0.8, 3.0) | 6.3 (3.9, 10.4) | 7.0 (5.0, 10.0) | 2.9 (0.7, 11.5) | 3.9 (1.3, 12.2) | 3.3 (1.9, 5.6) | 1.2 (1.0, 1.5) |
| CED | | | | | | | | 4.1 (3.8, 4.4) | 4.4 (4.0, 4.8) | 3.9 (3.4, 4.5) | 8.1 (6.5, 10.1) | 3.6 (2.9, 4.4) | 1.1 (0.9, 1.4) | 1.6 (1.2, 2.1) | 1.8 (1.6, 2.0) | 1.8 (1.5, 2.1) | 4.3 (3.7, 5.2) | 6.0 (5.4, 6.7) | 4.7 (3.4, 6.5) | 1.5 (0.9, 2.6) | 2.6 (2.2, 3.1) | 1.4 (1.3, 1.4) |
| UC | | | | | | | | | 41.4 (40.1, 42.8) | 6.6 (6.0, 7.2) | 6.2 (5.2, 7.5) | 31.1(29.2, 33.1) | 1.2 (1.0, 1.4) | 1.2 (1.0, 1.5) | 1.6 (1.5, 1.8) | 5.9 (5.4, 6.3) | 2.4 (2.0, 2.8) | 2.2 (1.9, 2.5) | 1.8 (1.3, 2.7) | 2.5 (1.8, 3.4) | 1.9 (1.7, 2.3) | 0.9 (0.8, 0.9) |
| CRD | | | | | | | | | | 4.0 (3.5, 4.6) | 3.1 (2.2, 4.3) | 14.9(13.5, 16.5) | 1.3 (1.1, 1.6) | 1.0 (0.8, 1.4) | 1.8 (1.6, 1.9) | 9.9 (9.2, 10.7) | 2.2 (1.8, 2.8) | 2.5 (2.2, 3.0) | 0.9 (0.4, 1.7) | 2.0 (1.3, 3.0) | 1.7 (1.4, 2.1) | 1.3 (1.2, 1.3) |
| AIH | | | | | | | | | | | 104.6 (94.5, 115.9) | 27.4(24.6, 30.4) | 1.3 (1.0, 1.6) | 3.6 (2.8, 4.6) | 2.4 (2.1, 2.7) | 2.2 (1.8, 2.7) | 9.9 (8.5, 11.6) | 4.9 (4.1, 5.7) | 4.6 (3.0, 7.0) | 2.9 (1.8, 4.9) | 2.0 (1.5, 2.6) | 1.0 (0.9, 1.0) |
| PBC | | | | | | | | | | | | 43.6 (36.4, 52.2) | 2.4 (1.6, 3.6) | 3.0 (1.6, 5.4) | 4.5 (3.7, 5.4) | 1.6 (0.9, 2.8) | 15.5(11.8, 20.3) | 33.1(28.6, 38.4) | 9.3 (4.8, 17.9) | 0.0 (0.0, 0.0) | 2.9 (1.7, 4.7) | 0.9 (0.7, 1.1) |
| PSC | | | | | | | | | | | | | 1.4 (1.0, 2.0) | 2.4 (1.6, 3.6) | 2.0 (1.6, 2.3) | 2.0 (1.5, 2.7) | 3.9 (2.8, 5.4) | 3.5 (2.7, 4.6) | 2.8 (1.3, 5.8) | 2.5 (1.2, 5.3) | 2.8 (2.0, 3.8) | 1.1 (1.0, 1.2) |
| MS | | | | | | | | | | | | | | 5.5 (4.7, 6.5) | 0.9 (0.8, 1.0) | 1.4 (1.1, 1.7) | 2.6 (2.1, 3.2) | 2.5 (2.1, 2.9) | 1.5 (0.9, 2.7) | 0.7 (0.3, 1.5) | 1.5 (1.2, 1.9) | 1.0 (1.0, 1.1) |
| MG | | | | | | | | | | | | | | | 2.8 (2.5, 3.2) | 1.7 (1.3, 2.4) | 4.7 (3.5, 6.2) | 3.6 (2.8, 4.5) | 15.0(11.1, 20.3) | 1.8 (0.8, 4.0) | 1.1 (0.7, 1.7) | 1.1 (1.0, 1.2) |
| RA | | | | | | | | | | | | | | | | 8.3 (7.9, 8.8) | 17.5(16.4, 18.7) | 24.9(23.9, 26.0) | 13.6(12.0, 15.6) | 8.1 (6.9, 9.4) | 1.9 (1.7, 2.2) | 0.9 (0.8, 0.9) |
| AS | | | | | | | | | | | | | | | | | 4.3 (3.5, 5.2) | 5.9 (5.2, 6.7) | 2.8 (1.8, 4.4) | 2.2 (1.4, 3.7) | 2.2 (1.7, 2.7) | 0.9 (0.9, 1.0) |
| SLE | | | | | | | | | | | | | | | | | | 77.9 (73.0, 83.1) | 65.9(56.3, 77.2) | 23.9(18.9, 30.2) | 2.9 (2.2, 3.8) | 1.2 (1.1, 1.3) |
| SJS | | | | | | | | | | | | | | | | | | | 28.6 (24.1, 34.0) | 6.7 (4.8, 9.2) | 4.0 (3.3, 4.7) | 1.0 (1.0, 1.1) |
| IIM | | | | | | | | | | | | | | | | | | | | 29.4 (19.8, 43.8) | 6.2 (4.3, 9.1) | 0.8 (0.7, 1.0) |
| GPA | | | | | | | | | | | | | | | | | | | | | 5.2 (3.6, 7.7) | 0.9 (0.7, 1.1) |
| SAR | | | | | | | | | | | | | | | | | | | | | | 1.3 (1.2, 1.4) |

**Supplemental Table S4B.** Adjusted odds ratios for co-occurrence of autoimmune diseases in individuals. 95% confidence intervals are given within parentheses. Adjusted for sex and age.

| | GD | T1D | ADD | PSO | PSA | DH | CED | UC | CRD | AIH | PBC | PSC | MS | MG | RA | AS | SLE | SJS | IIM | GPA | SAR | APP |
|---|---|---|---|---|---|---|---|---|---|---|---|---|---|---|---|---|---|---|---|---|---|---|
| HT | 0.6 (0.6, 0.7) | 4.1 (3.9, 4.2) | 15.4(13.2, 17.9) | 1.4 (1.3, 1.4) | 1.5 (1.4, 1.6) | 2.3 (2.0, 2.7) | 2.2 (2.2, 2.3) | 1.1 (1.0, 1.1) | 0.9 (0.9, 1.0) | 1.5 (1.4, 1.6) | 2.0 (1.8, 2.2) | 1.2 (1.0, 1.3) | 1.0 (1.0, 1.1) | 1.5 (1.3, 1.6) | 1.3 (1.3, 1.4) | 1.1 (1.0, 1.2) | 1.8 (1.6, 1.9) | 2.2 (2.1, 2.3) | 1.7 (1.5, 2.0) | 1.5 (1.3, 1.7) | 1.6 (1.5, 1.7) | 1.2 (1.1, 1.2) |
| GD | | 3.7 (3.5, 4.0) | 9.7(7.5, 12.4) | 1.2 (1.1, 1.3) | 1.5 (1.3, 1.6) | 2.1 (1.4, 3.0) | 2.5 (2.3, 2.7) | 1.4 (1.3, 1.6) | 1.4 (1.2, 1.6) | 1.4 (1.2, 1.7) | 1.5 (1.1, 2.0) | 1.6 (1.2, 2.0) | 0.9 (0.8, 1.1) | 1.9 (1.6, 2.4) | 1.5 (1.4, 1.7) | 1.0 (0.9, 1.3) | 1.6 (1.4, 2.0) | 1.8 (1.6, 2.0) | 1.8 (1.3, 2.6) | 2.7 (2.0, 3.6) | 2.0 (1.7, 2.3) | 1.2 (1.1, 1.2) |
| T1D | | | 21.3(17.9, 25.3) | 1.5 (1.4, 1.6) | 1.4 (1.2, 1.6) | 5.3 (4.2, 6.7) | 6.3 (5.9, 6.7) | 1.4 (1.3, 1.6) | 0.9 (0.8, 1.1) | 2.1 (1.9, 2.5) | 2.3 (1.7, 3.2) | 2.6 (2.2, 3.1) | 1.1 (1.0, 1.3) | 1.8 (1.5, 2.2) | 2.3 (2.2, 2.5) | 0.9 (0.8, 1.1) | 1.3 (1.0, 1.6) | 1.4 (1.2, 1.6) | 1.6 (1.0, 2.3) | 1.6 (1.1, 2.3) | 2.1 (1.8, 2.4) | 1.3 (1.2, 1.3) |
| ADD | | | | 1.3 (0.8, 2.0) | 1.4 (0.7, 2.9) | 2.7(0.4, 19.3) | 9.2(6.7, 12.7) | 1.0 (0.5, 2.0) | 1.6 (0.8, 3.2) | 3.6 (1.7, 7.4) | 3.4(0.8, 13.6) | 1.5 (0.4, 6.1) | 0.9 (0.3, 2.4) | 3.8 (1.7, 8.5) | 1.3 (0.8, 2.2) | 0.9 (0.3, 2.7) | 1.8 (0.6, 5.6) | 2.5 (1.2, 5.0) | 0.0 (0.0, 0.0) | 0.0 (0.0, 0.0) | 3.3 (1.7, 6.6) | 1.8 (1.5, 2.2) |
| PSO | | | | | 66.8(65.1, 68.5) | 3.2 (2.6, 4.0) | 1.9 (1.7, 2.1) | 1.7 (1.6, 1.9) | 2.1 (2.0, 2.3) | 1.8 (1.7, 2.0) | 2.1 (1.7, 2.6) | 1.5 (1.3, 1.8) | 1.1 (1.0, 1.3) | 1.3 (1.1, 1.5) | 2.6 (2.5, 2.7) | 3.0 (2.8, 3.3) | 1.8 (1.6, 2.1) | 2.4 (2.2, 2.6) | 2.3 (1.9, 2.9) | 1.6 (1.2, 2.1) | 1.8 (1.6, 2.0) | 1.1 (1.1, 1.1) |
| PSA | | | | | | 2.0 (1.3, 3.1) | 2.3 (2.1, 2.7) | 2.0 (1.8, 2.2) | 2.8 (2.5, 3.1) | 2.0 (1.7, 2.3) | 3.2 (2.4, 4.3) | 1.7 (1.3, 2.2) | 0.8 (0.7, 1.0) | 1.8 (1.4, 2.3) | 13.5(12.9, 14.0) | 10.9(10.2, 11.7) | 4.0 (3.4, 4.7) | 5.6 (5.1, 6.2) | 4.1 (3.0, 5.5) | 3.3 (2.4, 4.5) | 2.2 (1.8, 2.6) | 1.3 (1.2, 1.3) |
| DH | | | | | | | 141.2(127. 2, 156.6) | 2.6 (1.9, 3.7) | 2.5 (1.6, 3.8) | 3.7 (2.4, 5.8) | 6.0(2.9, 12.7) | 3.1 (1.7, 5.8) | 1.0 (0.5, 2.0) | 1.4 (0.6, 3.3) | 1.7 (1.2, 2.3) | 1.4 (0.7, 2.7) | 5.7 (3.5, 9.3) | 5.6 (3.9, 8.0) | 2.1 (0.5, 8.6) | 2.8 (0.9, 8.7) | 3.1 (1.8, 5.3) | 1.4 (1.2, 1.8) |
| CED | | | | | | | | 4.2 (3.9, 4.6) | 4.4 (4.0, 4.8) | 4.3 (3.7, 4.9) | 7.5 (6.0, 9.3) | 3.8 (3.1, 4.6) | 1.0 (0.9, 1.3) | 1.8 (1.4, 2.4) | 1.7 (1.5, 1.9) | 2.0 (1.7, 2.4) | 3.8 (3.2, 4.5) | 5.4 (4.9, 6.1) | 4.7 (3.4, 6.5) | 1.6 (0.9, 2.7) | 2.9 (2.4, 3.4) | 1.4 (1.3, 1.5) |
| UC | | | | | | | | | 41.3(39.9, 42.7) | 6.5 (5.9, 7.1) | 6.5 (5.4, 7.8) | 31.9(29.9, 34.0) | 1.2 (1.0, 1.4) | 1.2 (1.0, 1.5) | 1.7 (1.5, 1.8) | 5.7 (5.3, 6.1) | 2.4 (2.1, 2.9) | 2.3 (2.0, 2.6) | 1.9 (1.3, 2.7) | 2.5 (1.8, 3.3) | 1.9 (1.6, 2.2) | 0.9 (0.8, 0.9) |
| CRD | | | | | | | | | | 4.0 (3.5, 4.5) | 2.9 (2.1, 4.1) | 15.4(13.9, 17.0) | 1.3 (1.1, 1.5) | 1.0 (0.8, 1.4) | 1.7 (1.6, 1.9) | 10.0(9.2, 10.7) | 2.1 (1.7, 2.7) | 2.4 (2.0, 2.8) | 0.8 (0.4, 1.7) | 2.0 (1.3, 3.0) | 1.7 (1.4, 2.1) | 1.3 (1.2, 1.4) |
| AIH | | | | | | | | | | | 79.2(71.1, 88.2) | 19.7(17.7, 22.0) | 1.2 (0.9, 1.5) | 2.6 (2.0, 3.4) | 1.8 (1.6, 2.0) | 1.9 (1.5, 2.4) | 8.8(7.5, 10.3) | 3.7 (3.2, 4.4) | 3.4 (2.2, 5.2) | 2.1 (1.3, 3.5) | 1.8 (1.4, 2.4) | 1.1 (1.0, 1.2) |
| PBC | | | | | | | | | | | | 29.7(24.6, 35.9) | 1.7 (1.1, 2.6) | 2.3 (1.3, 4.1) | 2.3 (1.9, 2.8) | 1.7 (1.0, 3.0) | 8.6(6.5, 11.3) | 14.5(12.4, 16.8) | 5.6(2.9, 10.9) | 0.0 (0.0, 0.0) | 2.9 (1.7, 4.7) | 1.2 (1.0, 1.4) |
| PSC | | | | | | | | | | | | | 1.4 (1.0, 1.9) | 1.6 (1.1, 2.5) | 1.3 (1.1, 1.6) | 1.8 (1.3, 2.4) | 3.4 (2.5, 4.8) | 2.5 (1.9, 3.3) | 1.9 (0.9, 4.0) | 1.7 (0.8, 3.5) | 2.5 (1.9, 3.5) | 1.4 (1.2, 1.5) |
| MS | | | | | | | | | | | | | | 5.5 (4.7, 6.4) | 0.7 (0.6, 0.8) | 1.5 (1.2, 1.8) | 1.9 (1.5, 2.4) | 1.7 (1.5, 2.1) | 1.3 (0.8, 2.4) | 0.7 (0.3, 1.5) | 1.5 (1.2, 2.0) | 1.1 (1.0, 1.1) |
| MG | | | | | | | | | | | | | | | 2.2 (1.9, 2.5) | 1.5 (1.1, 2.0) | 4.6 (3.5, 6.1) | 3.2 (2.5, 4.0) | 11.4(8.4, 15.4) | 1.3 (0.6, 2.8) | 1.0 (0.6, 1.6) | 1.3 (1.2, 1.4) |
| RA | | | | | | | | | | | | | | | | 8.7 (8.2, 9.2) | 11.9(11.1, 12.8) | 13.9(13.3, 14.6) | 9.4(8.2, 10.8) | 5.9 (5.0, 6.9) | 1.9 (1.6, 2.1) | 1.1 (1.0, 1.1) |
| AS | | | | | | | | | | | | | | | | | 5.1 (4.2, 6.2) | 7.2 (6.3, 8.2) | 2.6 (1.7, 4.2) | 2.0 (1.2, 3.2) | 2.0 (1.6, 2.5) | 1.0 (0.9, 1.0) |
| SLE | | | | | | | | | | | | | | | | | | 45.4(42.3, 48.7) | 52.2(44.2, 61.5) | 21.2(16.7, 27.0) | 3.1 (2.3, 4.1) | 1.3 (1.2, 1.5) |
| SJS | | | | | | | | | | | | | | | | | | | 18.8(15.7, 22.5) | 4.9 (3.6, 6.9) | 4.1 (3.4, 4.9) | 1.3 (1.2, 1.4) |
| IIM | | | | | | | | | | | | | | | | | | | | 21.0(14.1, 31.4) | 5.9 (4.1, 8.6) | 1.0 (0.8, 1.3) |
| GPA | | | | | | | | | | | | | | | | | | | | | 4.8 (3.3, 7.1) | 1.1 (0.9, 1.3) |
| SAR | | | | | | | | | | | | | | | | | | | | | | 1.3 (1.2, 1.4) |

**Supplemental Table S5A.** Unadjusted odds ratios for aggregation and coaggregation across full siblings**.**
95% confidence intervals are given within parentheses. NA, not applicable – no observed sibling pair with the disease combination.

| | HT | GD | T1D | ADD | PSO | PSA | DH | CED | UC | CRD | AIH | PBC | PSC | MS | MG | RA | AS | SLE | SJS | IIM | GPA | SAR | APP |
|---|---|---|---|---|---|---|---|---|---|---|---|---|---|---|---|---|---|---|---|---|---|---|---|
| HT | 2.7 (2.6, 2.7) | 1.6 (1.6, 1.7) | 1.5 (1.4, 1.5) | 2.2 (1.9, 2.7) | 1.2 (1.2, 1.2) | 1.3 (1.2, 1.4) | 1.5 (1.3, 1.7) | 1.3 (1.3, 1.4) | 1.0 (1.0, 1.1) | 1.0 (0.9, 1.0) | 1.3 (1.2, 1.4) | 1.5 (1.3, 1.8) | 1.3 (1.2, 1.4) | 1.1 (1.0, 1.1) | 1.2 (1.2, 1.4) | 1.5 (1.4, 1.5) | 1.1 (1.0, 1.1) | 1.4 (1.3, 1.5) | 1.5 (1.4, 1.6) | 1.3 (1.1, 1.5) | 1.4 (1.3, 1.7) | 1.2 (1.1, 1.3) | 1.0 (0.9, 1.0) |
| GD | | 3.8 (3.5, 4.1) | 1.6 (1.5, 1.8) | 2.5 (1.7, 3.8) | 1.1 (1.0, 1.1) | 1.1 (1.0, 1.3) | 2.2 (1.6, 2.9) | 1.5 (1.4, 1.7) | 1.1 (1.0, 1.2) | 1.2 (1.1, 1.3) | 1.4 (1.2, 1.7) | 1.5 (1.1, 2.1) | 1.0 (0.8, 1.3) | 1.1 (0.9, 1.2) | 1.2 (1.0, 1.4) | 1.4 (1.3, 1.5) | 1.0 (0.9, 1.2) | 1.5 (1.3, 1.8) | 1.4 (1.2, 1.6) | 1.5 (1.1, 2.2) | 1.5 (1.1, 2.0) | 1.1 (1.0, 1.3) | 1.0 (1.0, 1.1) |
| T1D | | | 8.7 (8.3, 9.3) | 3.9 (2.9, 5.4) | 1.0 (1.0, 1.1) | 1.2 (1.1, 1.4) | 1.7 (1.2, 2.3) | 1.9 (1.7, 2.1) | 1.1 (1.0, 1.2) | 0.9 (0.8, 1.0) | 1.1 (0.9, 1.3) | 1.6 (1.1, 2.1) | 1.3 (1.0, 1.5) | 1.2 (1.1, 1.4) | 1.1 (0.9, 1.4) | 1.5 (1.4, 1.7) | 1.0 (0.9, 1.2) | 1.4 (1.2, 1.7) | 1.3 (1.1, 1.5) | 1.3 (0.9, 1.9) | 1.2 (0.8, 1.7) | 1.5 (1.2, 1.7) | 1.1 (1.0, 1.1) |
| ADD | | | | 99.3 (58.7, 168.0) | 0.7 (0.4, 1.1) | 1.0 (0.5, 1.9) | 1.8 (0.2, 12.4) | 2.8 (1.7, 4.4) | 0.4 (0.2, 1.0) | 0.9 (0.4, 1.9) | 2.1 (0.9, 4.8) | 1.3 (0.2, 9.0) | 0.5 (0.1, 3.7) | 1.1 (0.4, 2.6) | 0.4 (0.1, 3.1) | 1.4 (0.9, 2.1) | 1.2 (0.5, 2.6) | 1.3 (0.4, 4.2) | 1.7 (0.8, 3.5) | 1.6 (0.2, 11.7) | NA | 2.8 (1.5, 5.3) | 1.1 (0.9, 1.4) |
| PSO | | | | | 3.4 (3.3, 3.6) | 3.6 (3.5, 3.8) | 1.1 (0.8, 1.4) | 1.2 (1.1, 1.4) | 1.2 (1.1, 1.2) | 1.3 (1.2, 1.4) | 1.4 (1.2, 1.5) | 1.7 (1.4, 2.1) | 1.3 (1.1, 1.5) | 1.1 (1.0, 1.2) | 1.2 (1.0, 1.4) | 1.3 (1.3, 1.4) | 1.5 (1.4, 1.6) | 1.3 (1.1, 1.5) | 1.4 (1.3, 1.5) | 1.3 (1.0, 1.7) | 1.4 (1.1, 1.8) | 1.3 (1.1, 1.4) | 1.0 (0.9, 1.0) |
| PSA | | | | | | 6.1 (5.6, 6.6) | 1.2 (0.7, 1.9) | 1.2 (1.0, 1.4) | 1.2 (1.1, 1.4) | 1.2 (1.1, 1.4) | 1.5 (1.2, 1.8) | 1.8 (1.3, 2.5) | 1.3 (1.0, 1.6) | 1.2 (1.0, 1.4) | 1.2 (1.0, 1.6) | 1.9 (1.7, 2.0) | 2.1 (1.8, 2.4) | 1.7 (1.4, 2.1) | 1.7 (1.4, 2.0) | 1.1 (0.6, 1.8) | 1.3 (0.9, 2.0) | 1.2 (1.0, 1.5) | 1.0 (1.0, 1.1) |
| DH | | | | | | | 14.6 (7.4, 28.6) | 4.6 (3.5, 6.2) | 1.6 (1.1, 2.3) | 1.0 (0.6, 1.7) | 0.9 (0.4, 2.0) | 2.6 (1.0, 6.9) | 1.3 (0.6, 3.2) | 0.8 (0.4, 1.6) | 2.0 (1.1, 3.9) | 1.8 (1.3, 2.3) | 0.8 (0.4, 1.6) | 2.1 (1.1, 4.0) | 2.6 (1.7, 3.9) | 1.7 (0.4, 6.7) | 0.8 (0.1, 5.4) | 2.5 (1.5, 4.0) | 1.0 (0.9, 1.2) |
| CED | | | | | | | | 11.6 (10.6, 12.7) | 1.3 (1.2, 1.5) | 1.3 (1.1, 1.5) | 1.3 (1.0, 1.6) | 1.4 (0.9, 2.1) | 1.0 (0.8, 1.4) | 1.0 (0.9, 1.3) | 0.9 (0.7, 1.3) | 1.1 (1.0, 1.3) | 0.9 (0.7, 1.1) | 1.7 (1.4, 2.2) | 1.4 (1.1, 1.7) | 1.4 (0.8, 2.3) | 1.3 (0.8, 2.0) | 1.5 (1.3, 1.8) | 1.1 (1.0, 1.1) |
| UC | | | | | | | | | 4.6 (4.3, 4.9) | 2.9 (2.7, 3.1) | 1.3 (1.1, 1.5) | 1.6 (1.2, 2.1) | 1.8 (1.5, 2.1) | 1.0 (0.9, 1.2) | 1.2 (1.0, 1.4) | 1.2 (1.1, 1.3) | 1.7 (1.5, 1.9) | 1.1 (0.9, 1.3) | 1.3 (1.2, 1.5) | 1.1 (0.7, 1.6) | 1.4 (1.0, 1.9) | 1.3 (1.1, 1.5) | 0.9 (0.8, 0.9) |
| CRD | | | | | | | | | | 7.5 (6.8, 8.3) | 1.3 (1.1, 1.6) | 0.7 (0.4, 1.2) | 1.7 (1.3, 2.1) | 1.2 (1.0, 1.4) | 1.1 (0.8, 1.4) | 1.2 (1.1, 1.3) | 2.1 (1.8, 2.4) | 1.1 (0.8, 1.5) | 1.3 (1.0, 1.5) | 1.2 (0.7, 2.0) | 1.3 (0.8, 2.1) | 1.2 (1.0, 1.5) | 0.9 (0.9, 1.0) |
| AIH | | | | | | | | | | | 3.1 (2.4, 4.0) | 4.3 (3.0, 6.1) | 1.8 (1.3, 2.5) | 1.1 (0.9, 1.4) | 1.6 (1.1, 2.1) | 1.5 (1.3, 1.7) | 1.3 (1.1, 1.7) | 1.9 (1.4, 2.5) | 1.6 (1.3, 2.0) | 1.8 (1.1, 3.2) | 1.4 (0.8, 2.5) | 1.1 (0.9, 1.5) | 0.9 (0.8, 1.0) |
| PBC | | | | | | | | | | | | 26.9 (18.2, 39.8) | 2.7 (1.5, 4.7) | 1.8 (1.3, 2.7) | 0.5 (0.2, 1.5) | 1.6 (1.3, 2.0) | 0.9 (0.5, 1.6) | 2.7 (1.6, 4.3) | 2.3 (1.6, 3.4) | 1.2 (0.3, 4.9) | 1.1 (0.3, 4.4) | 1.7 (1.0, 2.7) | 0.8 (0.7, 1.0) |
| PSC | | | | | | | | | | | | | 3.0 (1.9, 4.6) | 1.2 (0.9, 1.6) | 1.3 (0.8, 2.1) | 1.6 (1.4, 1.8) | 1.2 (0.9, 1.7) | 1.3 (0.8, 2.0) | 1.6 (1.1, 2.2) | 1.2 (0.5, 3.0) | 1.1 (0.5, 2.7) | 1.4 (1.0, 1.9) | 0.8 (0.8, 0.9) |
| MS | | | | | | | | | | | | | | 7.5 (6.7, 8.3) | 1.0 (0.7, 1.3) | 1.1 (1.0, 1.2) | 1.3 (1.1, 1.5) | 1.3 (1.0, 1.7) | 1.4 (1.2, 1.7) | 1.1 (0.6, 1.9) | 0.9 (0.5, 1.6) | 1.5 (1.2, 1.8) | 1.0 (1.0, 1.1) |
| MG | | | | | | | | | | | | | | | 5.4 (4.0, 7.3) | 1.7 (1.5, 2.0) | 1.4 (1.0, 1.8) | 1.4 (0.9, 2.1) | 1.9 (1.5, 2.5) | 1.9 (0.9, 3.9) | 0.8 (0.3, 2.0) | 1.1 (0.8, 1.6) | 1.0 (0.9, 1.0) |
| RA | | | | | | | | | | | | | | | | 4.1 (3.9, 4.3) | 1.8 (1.7, 2.0) | 2.3 (2.1, 2.6) | 2.5 (2.3, 2.7) | 1.8 (1.4, 2.3) | 2.6 (2.1, 3.3) | 1.4 (1.2, 1.5) | 0.9 (0.9, 0.9) |
| AS | | | | | | | | | | | | | | | | | 15.5 (14.2, 16.9) | 1.6 (1.2, 2.0) | 1.4 (1.2, 1.8) | 1.1 (0.6, 2.3) | 0.5 (0.2, 1.1) | 1.3 (1.0, 1.6) | 1.0 (0.9, 1.0) |
| SLE | | | | | | | | | | | | | | | | | | 11.9 (9.5, 14.9) | 4.1 (3.4, 5.0) | 3.3 (1.9, 5.5) | 2.1 (1.2, 3.9) | 1.3 (0.9, 1.8) | 1.0 (1.0, 1.1) |
| SJS | | | | | | | | | | | | | | | | | | | 6.3 (5.4, 7.5) | 2.6 (1.7, 4.0) | 1.4 (0.9, 2.4) | 1.3 (1.0, 1.6) | 0.9 (0.8, 0.9) |
| IIM | | | | | | | | | | | | | | | | | | | | 8.0 (3.3, 19.1) | 1.4 (0.4, 5.7) | 1.4 (0.7, 2.5) | 1.1 (0.9, 1.3) |
| GPA | | | | | | | | | | | | | | | | | | | | | 11.6 (5.1, 26.9) | 1.1 (0.6, 2.1) | 0.9 (0.7, 1.0) |
| SAR | | | | | | | | | | | | | | | | | | | | | | 4.9 (4.0, 6.0) | 1.1 (1.0, 1.1) |
| APP | | | | | | | | | | | | | | | | | | | | | | | 1.7 (1.7, 1.7) |

**Supplemental Table S5B.** Adjusted odds ratios for aggregation and coaggregation across full siblings**.** Adjusted for sex and age. 95% confidence intervals are given within parentheses. NA, not applicable – no observed sibling pair with the disease combination.

| | HT | GD | T1D | ADD | PSO | PSA | DH | CED | UC | CRD | AIH | PBC | PSC | MS | MG | RA | AS | SLE | SJS | IIM | GPA | SAR | APP |
|---|---|---|---|---|---|---|---|---|---|---|---|---|---|---|---|---|---|---|---|---|---|---|---|
| HT | 2.6 (2.6, 2.7) | 1.6 (1.5, 1.6) | 1.5 (1.5, 1.6) | 2.3 (1.9, 2.8) | 1.1 (1.1, 1.2) | 1.2 (1.2, 1.3) | 1.3 (1.1, 1.5) | 1.3 (1.3, 1.4) | 1.0 (1.0, 1.1) | 1.0 (0.9, 1.0) | 1.2 (1.1, 1.3) | 1.3 (1.1, 1.5) | 1.1 (1.0, 1.2) | 1.0 (1.0, 1.1) | 1.1 (1.0, 1.2) | 1.3 (1.2, 1.3) | 1.0 (1.0, 1.1) | 1.3 (1.2, 1.4) | 1.3 (1.2, 1.4) | 1.1 (1.0, 1.3) | 1.2 (1.1, 1.4) | 1.2 (1.1, 1.3) | 1.0 (1.0, 1.1) |
| GD | | 3.7 (3.4, 4.0) | 1.7 (1.5, 1.8) | 2.5 (1.7, 3.8) | 1.0 (1.0, 1.1) | 1.1 (1.0, 1.2) | 2.0 (1.5, 2.7) | 1.6 (1.4, 1.7) | 1.1 (1.0, 1.2) | 1.2 (1.0, 1.3) | 1.3 (1.1, 1.5) | 1.3 (1.0, 1.8) | 0.9 (0.7, 1.1) | 1.0 (0.9, 1.2) | 1.1 (0.9, 1.3) | 1.3 (1.2, 1.4) | 1.0 (0.8, 1.1) | 1.5 (1.2, 1.7) | 1.3 (1.1, 1.4) | 1.4 (1.0, 2.0) | 1.3 (0.9, 1.8) | 1.1 (0.9, 1.3) | 1.1 (1.0, 1.1) |
| T1D | | | 8.7 (8.2, 9.2) | 3.9 (2.9, 5.3) | 1.0 (1.0, 1.1) | 1.2 (1.1, 1.4) | 1.7 (1.3, 2.4) | 1.9 (1.7, 2.1) | 1.1 (1.0, 1.2) | 0.9 (0.8, 1.0) | 1.1 (0.9, 1.3) | 1.6 (1.2, 2.2) | 1.3 (1.1, 1.6) | 1.2 (1.1, 1.4) | 1.1 (0.9, 1.4) | 1.6 (1.5, 1.7) | 1.0 (0.9, 1.2) | 1.4 (1.2, 1.7) | 1.3 (1.2, 1.6) | 1.4 (0.9, 2.0) | 1.2 (0.9, 1.8) | 1.4 (1.2, 1.7) | 1.1 (1.0, 1.1) |
| ADD | | | | 98.6(58.2 , 167.0) | 0.7 (0.4, 1.1) | 1.0 (0.5, 1.9) | 1.7(0.2, 12.3) | 2.8 (1.8, 4.5) | 0.4 (0.2, 1.0) | 0.9 (0.4, 1.9) | 2.0 (0.9, 4.8) | 1.3 (0.2, 9.0) | 0.5 (0.1, 3.6) | 1.1 (0.4, 2.6) | 0.4 (0.1, 3.1) | 1.4 (0.9, 2.1) | 1.2 (0.5, 2.6) | 1.3 (0.4, 4.2) | 1.7 (0.8, 3.5) | 1.6(0.2, 11.6) | 0.0 (0.0, 0.0) | 2.8 (1.5, 5.2) | 1.1 (0.9, 1.4) |
| PSO | | | | | 3.3 (3.2, 3.4) | 3.5 (3.3, 3.6) | 1.0 (0.7, 1.3) | 1.3 (1.2, 1.4) | 1.1 (1.1, 1.2) | 1.3 (1.2, 1.4) | 1.3 (1.1, 1.4) | 1.5 (1.2, 1.8) | 1.1 (1.0, 1.3) | 1.0 (0.9, 1.1) | 1.1 (0.9, 1.3) | 1.2 (1.1, 1.2) | 1.4 (1.3, 1.6) | 1.2 (1.0, 1.4) | 1.2 (1.1, 1.4) | 1.2 (0.9, 1.5) | 1.2 (1.0, 1.6) | 1.2 (1.1, 1.4) | 1.0 (1.0, 1.0) |
| PSA | | | | | | 5.7 (5.3, 6.2) | 1.1 (0.7, 1.8) | 1.3 (1.1, 1.5) | 1.2 (1.1, 1.3) | 1.2 (1.1, 1.4) | 1.3 (1.1, 1.6) | 1.6 (1.1, 2.2) | 1.2 (0.9, 1.5) | 1.1 (1.0, 1.3) | 1.1 (0.9, 1.4) | 1.7 (1.6, 1.8) | 2.0 (1.8, 2.2) | 1.6 (1.3, 2.0) | 1.5 (1.3, 1.8) | 1.0 (0.6, 1.7) | 1.2 (0.8, 1.8) | 1.2 (1.0, 1.4) | 1.1 (1.0, 1.1) |
| DH | | | | | | | 11.7(5.9, 22.9) | 4.7 (3.5, 6.2) | 1.6 (1.1, 2.2) | 1.0 (0.6, 1.7) | 0.7 (0.3, 1.7) | 2.0 (0.7, 5.3) | 1.0 (0.4, 2.4) | 0.8 (0.4, 1.5) | 1.6 (0.8, 3.1) | 1.4 (1.0, 1.8) | 0.7 (0.4, 1.5) | 1.9 (1.0, 3.6) | 2.0 (1.3, 3.1) | 1.4 (0.3, 5.5) | 0.6 (0.1, 4.2) | 2.4 (1.5, 3.8) | 1.2 (1.0, 1.4) |
| CED | | | | | | | | 11.2(10.2 , 12.2) | 1.3 (1.2, 1.5) | 1.3 (1.1, 1.5) | 1.3 (1.1, 1.7) | 1.4 (0.9, 2.2) | 1.0 (0.8, 1.4) | 1.1 (0.9, 1.3) | 1.0 (0.7, 1.3) | 1.2 (1.0, 1.3) | 0.9 (0.8, 1.2) | 1.7 (1.4, 2.2) | 1.4 (1.2, 1.7) | 1.4 (0.9, 2.4) | 1.3 (0.8, 2.0) | 1.6 (1.3, 1.9) | 1.1 (1.0, 1.1) |
| UC | | | | | | | | | 4.6 (4.2, 4.9) | 2.9 (2.7, 3.1) | 1.3 (1.1, 1.5) | 1.5 (1.1, 2.1) | 1.8 (1.5, 2.1) | 1.0 (0.9, 1.2) | 1.2 (1.0, 1.4) | 1.1 (1.1, 1.2) | 1.7 (1.5, 1.9) | 1.1 (0.9, 1.3) | 1.3 (1.1, 1.5) | 1.1 (0.7, 1.6) | 1.3 (0.9, 1.9) | 1.3 (1.1, 1.5) | 0.9 (0.8, 0.9) |
| CRD | | | | | | | | | | 7.5 (6.8, 8.2) | 1.3 (1.1, 1.6) | 0.7 (0.4, 1.2) | 1.7 (1.3, 2.1) | 1.1 (1.0, 1.4) | 1.1 (0.8, 1.4) | 1.1 (1.0, 1.3) | 2.0 (1.8, 2.3) | 1.1 (0.8, 1.4) | 1.2 (1.0, 1.5) | 1.2 (0.7, 1.9) | 1.3 (0.8, 2.1) | 1.2 (1.0, 1.5) | 0.9 (0.9, 1.0) |
| AIH | | | | | | | | | | | 2.6 (2.0, 3.3) | 3.3 (2.3, 4.7) | 1.4 (1.1, 2.0) | 1.0 (0.8, 1.3) | 1.3 (0.9, 1.7) | 1.2 (1.1, 1.3) | 1.2 (1.0, 1.5) | 1.7 (1.2, 2.2) | 1.3 (1.0, 1.6) | 1.5 (0.9, 2.6) | 1.1 (0.6, 2.0) | 1.1 (0.8, 1.4) | 1.0 (1.0, 1.1) |
| PBC | | | | | | | | | | | | 18.6(12.5 , 27.7) | 1.9 (1.1, 3.3) | 1.7 (1.2, 2.5) | 0.4 (0.1, 1.1) | 1.1 (0.9, 1.4) | 0.8 (0.4, 1.4) | 2.2 (1.4, 3.7) | 1.6 (1.1, 2.4) | 0.9 (0.2, 3.7) | 0.8 (0.2, 3.2) | 1.6 (1.0, 2.6) | 1.0 (0.9, 1.2) |
| PSC | | | | | | | | | | | | | 2.1 (1.3, 3.2) | 1.1 (0.8, 1.5) | 1.0 (0.6, 1.6) | 1.2 (1.0, 1.3) | 1.1 (0.8, 1.5) | 1.1 (0.7, 1.7) | 1.2 (0.8, 1.6) | 1.0 (0.4, 2.3) | 0.8 (0.3, 2.0) | 1.3 (0.9, 1.8) | 1.0 (0.9, 1.1) |
| MS | | | | | | | | | | | | | | 7.4 (6.6, 8.2) | 0.9 (0.7, 1.2) | 1.0 (0.9, 1.1) | 1.2 (1.0, 1.5) | 1.2 (1.0, 1.6) | 1.3 (1.1, 1.6) | 1.1 (0.6, 1.8) | 0.9 (0.5, 1.5) | 1.4 (1.1, 1.8) | 1.1 (1.0, 1.1) |
| MG | | | | | | | | | | | | | | | 4.3 (3.2, 5.8) | 1.3 (1.2, 1.5) | 1.2 (0.9, 1.6) | 1.2 (0.8, 1.9) | 1.5 (1.2, 1.9) | 1.5 (0.7, 3.2) | 0.6 (0.2, 1.6) | 1.0 (0.7, 1.5) | 1.1 (1.0, 1.2) |
| RA | | | | | | | | | | | | | | | | 3.1 (2.9, 3.3) | 1.7 (1.5, 1.8) | 2.0 (1.8, 2.3) | 1.9 (1.7, 2.1) | 1.4 (1.1, 1.8) | 2.0 (1.6, 2.4) | 1.3 (1.1, 1.4) | 1.0 (1.0, 1.1) |
| AS | | | | | | | | | | | | | | | | | 14.9(13.6 , 16.3) | 1.5 (1.1, 1.9) | 1.3 (1.1, 1.6) | 1.0 (0.5, 2.1) | 0.5 (0.2, 1.0) | 1.2 (1.0, 1.6) | 1.0 (1.0, 1.1) |
| SLE | | | | | | | | | | | | | | | | | | 11.3(8.9, 14.2) | 3.6 (3.0, 4.4) | 2.9 (1.7, 4.9) | 1.8 (1.0, 3.3) | 1.2 (0.9, 1.7) | 1.1 (1.0, 1.2) |
| SJS | | | | | | | | | | | | | | | | | | | 5.0 (4.2, 5.9) | 2.1 (1.3, 3.2) | 1.1 (0.6, 1.8) | 1.2 (0.9, 1.5) | 1.0 (1.0, 1.1) |
| IIM | | | | | | | | | | | | | | | | | | | | 6.5(2.7, 15.6) | 1.1 (0.3, 4.5) | 1.3 (0.7, 2.4) | 1.2 (1.0, 1.4) |
| GPA | | | | | | | | | | | | | | | | | | | | | 9.1(4.0, 21.0) | 1.0 (0.5, 2.0) | 1.0 (0.8, 1.2) |
| SAR | | | | | | | | | | | | | | | | | | | | | | 4.8 (3.9, 5.8) | 1.1 (1.0, 1.2) |
| APP | | | | | | | | | | | | | | | | | | | | | | | 1.6 (1.6, 1.6) |

**Supplemental Table S6A.** Unadjusted genetic correlations for combinations of autoimmune diseases**.** 95% confidence intervals are given within parentheses.

| | GD | T1D | ADD | PSO | PSA | DH | CED | UC | CRD | AIH | PBC | PSC | MS | MG | RA | AS | SLE | SJS | IIM | GPA | SAR | APP |
|---|---|---|---|---|---|---|---|---|---|---|---|---|---|---|---|---|---|---|---|---|---|---|
| HT | 0.40 (0.38, 0.42) | 0.25 (0.23, 0.26) | 0.33 (0.28, 0.39) | 0.15 (0.13, 0.16) | 0.18 (0.16, 0.20) | 0.21 (0.15, 0.27) | 0.16 (0.14, 0.18) | 0.03 (0.01, 0.05) | -0.02 (-0.04, 0.00) | 0.25 (0.21, 0.30) | 0.21 (0.17, 0.25) | 0.23 (0.16, 0.29) | 0.03 (0.01, 0.06) | 0.16 (0.12, 0.20) | 0.31 (0.30, 0.33) | 0.04 (0.02, 0.06) | 0.20 (0.17, 0.23) | 0.28 (0.26, 0.31) | 0.17 (0.10, 0.24) | 0.21 (0.15, 0.28) | 0.11 (0.09, 0.14) | -0.05 (-0.07, -0.04) |
| GD | | 0.26 (0.23, 0.29) | 0.33 (0.22, 0.43) | 0.05 (0.02, 0.08) | 0.06 (0.02, 0.11) | 0.37 (0.26, 0.49) | 0.20 (0.17, 0.24) | 0.07 (0.03, 0.11) | 0.09 (0.05, 0.13) | 0.27 (0.18, 0.35) | 0.17 (0.08, 0.26) | -0.00 (-0.12, 0.12) | 0.03 (-0.02, 0.08) | 0.09 (0.01, 0.18) | 0.24 (0.20, 0.27) | -0.00 (-0.05, 0.04) | 0.21 (0.14, 0.27) | 0.19 (0.13, 0.24) | 0.23 (0.09, 0.37) | 0.18 (0.07, 0.30) | 0.07 (0.01, 0.13) | 0.01 (-0.02, 0.04) |
| T1D | | | 0.37 (0.31, 0.44) | 0.02 (0.00, 0.05) | 0.09 (0.06, 0.12) | 0.34 (0.25, 0.44) | 0.24 (0.21, 0.27) | 0.04 (0.01, 0.07) | -0.04 (-0.07, -0.00) | 0.06 (-0.01, 0.12) | 0.14 (0.07, 0.20) | 0.13 (0.04, 0.22) | 0.08 (0.04, 0.11) | 0.04 (-0.02, 0.11) | 0.22 (0.19, 0.24) | 0.01 (-0.02, 0.04) | 0.13 (0.08, 0.17) | 0.12 (0.08, 0.16) | 0.12 (0.01, 0.22) | 0.07 (-0.03, 0.16) | 0.15 (0.11, 0.19) | 0.07 (0.05, 0.09) |
| ADD | | | | -0.13 (-0.24, -0.02) | -0.01 (-0.14, 0.13) | 0.13 (-0.20, 0.46) | 0.25 (0.16, 0.34) | -0.23 (-0.40, -0.07) | -0.03 (-0.18, 0.11) | 0.27 (0.04, 0.51) | 0.05 (-0.23, 0.32) | -0.23 (-0.69, 0.23) | 0.02 (-0.13, 0.16) | -0.22 (-0.57, 0.13) | 0.10 (0.01, 0.20) | 0.03 (-0.09, 0.16) | 0.07 (-0.12, 0.26) | 0.14 (-0.01, 0.30) | NA | NA | 0.28 (0.15, 0.41) | 0.07 (-0.01, 0.15) |
| PSO | | | | | 0.85 (0.83, 0.87) | 0.04 (-0.06, 0.13) | 0.11 (0.08, 0.13) | 0.09 (0.07, 0.12) | 0.15 (0.13, 0.18) | 0.26 (0.19, 0.32) | 0.24 (0.18, 0.30) | 0.19 (0.10, 0.28) | 0.03 (-0.00, 0.07) | 0.12 (0.06, 0.18) | 0.19 (0.16, 0.21) | 0.36 (0.33, 0.39) | 0.12 (0.07, 0.17) | 0.20 (0.16, 0.24) | 0.14 (0.03, 0.24) | 0.17 (0.08, 0.25) | 0.14 (0.09, 0.18) | -0.05 (-0.07, -0.02) |
| PSA | | | | | | 0.07 (-0.06, 0.19) | 0.07 (0.04, 0.11) | 0.10 (0.06, 0.14) | 0.10 (0.06, 0.14) | 0.24 (0.16, 0.32) | 0.20 (0.12, 0.28) | 0.16 (0.05, 0.27) | 0.07 (0.03, 0.12) | 0.11 (0.03, 0.19) | 0.35 (0.32, 0.38) | 0.29 (0.25, 0.32) | 0.23 (0.17, 0.28) | 0.25 (0.20, 0.30) | 0.02 (-0.12, 0.17) | 0.11 (-0.00, 0.23) | 0.08 (0.02, 0.14) | 0.01 (-0.02, 0.04) |
| DH | | | | | | | 0.55 (0.46, 0.64) | 0.21 (0.10, 0.31) | -0.01 (-0.15, 0.13) | -0.05 (-0.32, 0.23) | 0.28 (0.06, 0.49) | 0.15 (-0.19, 0.48) | -0.07 (-0.23, 0.09) | 0.30 (0.09, 0.51) | 0.26 (0.17, 0.35) | -0.06 (-0.20, 0.08) | 0.24 (0.08, 0.41) | 0.38 (0.25, 0.52) | 0.19 (-0.18, 0.57) | -0.09 (-0.52, 0.34) | 0.34 (0.20, 0.48) | 0.01 (-0.07, 0.10) |
| CED | | | | | | | | 0.12 (0.09, 0.16) | 0.09 (0.05, 0.13) | 0.13 (0.05, 0.21) | 0.09 (0.01, 0.18) | 0.02 (-0.09, 0.14) | 0.01 (-0.03, 0.06) | -0.03 (-0.11, 0.06) | 0.06 (0.03, 0.09) | -0.03 (-0.07, 0.01) | 0.19 (0.13, 0.24) | 0.13 (0.08, 0.18) | 0.13 (-0.00, 0.26) | 0.08 (-0.04, 0.19) | 0.15 (0.10, 0.21) | 0.04 (0.01, 0.07) |
| UC | | | | | | | | | 0.57 (0.54, 0.59) | 0.19 (0.11, 0.26) | 0.17 (0.10, 0.25) | 0.42 (0.31, 0.52) | 0.01 (-0.03, 0.06) | 0.09 (0.01, 0.17) | 0.09 (0.06, 0.12) | 0.23 (0.19, 0.26) | 0.03 (-0.03, 0.09) | 0.15 (0.10, 0.20) | 0.04 (-0.09, 0.18) | 0.14 (0.03, 0.25) | 0.12 (0.07, 0.17) | -0.13 (-0.16, -0.10) |
| CRD | | | | | | | | | | 0.16 (0.08, 0.24) | -0.11 (-0.22, 0.01) | 0.32 (0.22, 0.43) | 0.06 (0.01, 0.11) | 0.03 (-0.05, 0.12) | 0.08 (0.04, 0.11) | 0.26 (0.23, 0.30) | 0.04 (-0.03, 0.11) | 0.10 (0.05, 0.16) | 0.07 (-0.07, 0.22) | 0.11 (-0.01, 0.23) | 0.09 (0.03, 0.14) | -0.05 (-0.08, -0.02) |
| AIH | | | | | | | | | | | 0.71 (0.58, 0.84) | 0.52 (0.31, 0.73) | 0.05 (-0.05, 0.14) | 0.30 (0.14, 0.45) | 0.29 (0.22, 0.36) | 0.15 (0.07, 0.22) | 0.33 (0.22, 0.45) | 0.30 (0.20, 0.41) | 0.37 (0.12, 0.62) | 0.18 (-0.05, 0.41) | 0.08 (-0.04, 0.19) | -0.11 (-0.17, -0.05) |
| PBC | | | | | | | | | | | | 0.47 (0.27, 0.67) | 0.20 (0.11, 0.29) | -0.24 (-0.49, 0.02) | 0.19 (0.12, 0.25) | -0.04 (-0.14, 0.06) | 0.29 (0.18, 0.41) | 0.29 (0.19, 0.39) | 0.06 (-0.25, 0.38) | NA | 0.16 (0.05, 0.28) | -0.11 (-0.18, -0.05) |
| PSC | | | | | | | | | | | | | 0.10 (-0.03, 0.23) | 0.19 (-0.02, 0.41) | 0.35 (0.25, 0.44) | 0.09 (-0.02, 0.20) | 0.13 (-0.04, 0.31) | 0.29 (0.14, 0.44) | 0.13 (-0.25, 0.51) | 0.06 (-0.28, 0.40) | 0.18 (0.03, 0.34) | -0.21 (-0.30, -0.12) |
| MS | | | | | | | | | | | | | | -0.02 (-0.12, 0.08) | 0.04 (0.01, 0.08) | 0.08 (0.04, 0.13) | 0.09 (0.02, 0.16) | 0.15 (0.09, 0.21) | 0.04 (-0.12, 0.21) | -0.03 (-0.18, 0.12) | 0.16 (0.10, 0.22) | 0.03 (-0.00, 0.06) |
| MG | | | | | | | | | | | | | | | 0.32 (0.26, 0.38) | 0.12 (0.04, 0.20) | 0.14 (0.02, 0.26) | 0.33 (0.23, 0.43) | 0.32 (0.08, 0.55) | -0.11 (-0.39, 0.17) | 0.04 (-0.07, 0.16) | -0.05 (-0.10, 0.01) |
| RA | | | | | | | | | | | | | | | | 0.27 (0.24, 0.30) | 0.41 (0.36, 0.45) | 0.53 (0.49, 0.56) | 0.30 (0.19, 0.41) | 0.48 (0.38, 0.57) | 0.15 (0.11, 0.20) | -0.12 (-0.15, -0.10) |
| AS | | | | | | | | | | | | | | | | | 0.14 (0.08, 0.20) | 0.13 (0.08, 0.19) | 0.04 (-0.10, 0.18) | -0.20 (-0.36, -0.04) | 0.08 (0.03, 0.14) | -0.03 (-0.05, 0.00) |
| SLE | | | | | | | | | | | | | | | | | | 0.62 (0.56, 0.68) | 0.48 (0.31, 0.65) | 0.27 (0.11, 0.43) | 0.09 (-0.00, 0.17) | 0.01 (-0.03, 0.06) |
| SJS | | | | | | | | | | | | | | | | | | | 0.46 (0.29, 0.63) | 0.15 (-0.01, 0.30) | 0.11 (0.03, 0.18) | -0.12 (-0.16, -0.08) |
| IIM | | | | | | | | | | | | | | | | | | | | 0.14 (-0.25, 0.52) | 0.13 (-0.06, 0.32) | 0.06 (-0.03, 0.16) |
| GPA | | | | | | | | | | | | | | | | | | | | | 0.04 (-0.14, 0.21) | -0.12 (-0.21, -0.03) |
| SAR | | | | | | | | | | | | | | | | | | | | | | 0.05 (0.02, 0.09) |

**Supplemental Table S6B.** Adjusted genetic correlations for combinations of autoimmune diseases. 95% confidence intervals are given within parentheses. Adjusted for sex and age.

| | GD | T1D | ADD | PSO | PSA | DH | CED | UC | CRD | AIH | PBC | PSC | MS | MG | RA | AS | SLE | SJS | IIM | GPA | SAR | APP |
|---|---|---|---|---|---|---|---|---|---|---|---|---|---|---|---|---|---|---|---|---|---|---|
| HT | 0.38 (0.34, 0.41) | 0.27 (0.25, 0.29) | 0.35 (0.28, 0.43) | 0.10 (0.08, 0.12) | 0.15 (0.12, 0.18) | 0.14 (0.06, 0.23) | 0.17 (0.14, 0.20) | 0.02 (-0.01, 0.05) | -0.02 (-0.05, 0.01) | 0.15 (0.09, 0.21) | 0.11 (0.04, 0.17) | 0.07 (-0.02, 0.16) | 0.01 (-0.02, 0.05) | 0.06 (0.00, 0.12) | 0.20 (0.18, 0.23) | 0.01 (-0.02, 0.04) | 0.16 (0.12, 0.20) | 0.19 (0.15, 0.23) | 0.09 (-0.01, 0.19) | 0.11 (0.03, 0.20) | 0.12 (0.08, 0.17) | 0.07 (0.05, 0.09) |
| GD | | 0.26 (0.22, 0.30) | 0.34 (0.21, 0.48) | 0.02 (-0.03, 0.06) | 0.04 (-0.03, 0.10) | 0.46 (0.29, 0.64) | 0.22 (0.16, 0.27) | 0.06 (0.00, 0.12) | 0.08 (0.02, 0.14) | 0.22 (0.09, 0.35) | 0.13 (-0.00, 0.26) | -0.08 (-0.29, 0.12) | 0.01 (-0.06, 0.08) | 0.04 (-0.08, 0.17) | 0.19 (0.14, 0.24) | -0.02 (-0.09, 0.04) | 0.18 (0.09, 0.27) | 0.14 (0.06, 0.22) | 0.18 (-0.02, 0.39) | 0.15 (-0.02, 0.33) | 0.08 (-0.02, 0.18) | 0.07 (0.02, 0.11) |
| T1D | | | 0.38 (0.29, 0.47) | 0.03 (-0.01, 0.06) | 0.10 (0.06, 0.15) | 0.21 (0.08, 0.33) | 0.25 (0.21, 0.28) | 0.04 (0.00, 0.08) | -0.04 (-0.09, 0.01) | 0.07 (-0.03, 0.17) | 0.16 (0.06, 0.25) | 0.18 (0.03, 0.32) | 0.08 (0.03, 0.13) | 0.05 (-0.04, 0.15) | 0.26 (0.22, 0.30) | 0.01 (-0.04, 0.05) | 0.13 (0.06, 0.20) | 0.14 (0.08, 0.20) | 0.13 (-0.03, 0.28) | 0.08 (-0.06, 0.22) | 0.17 (0.10, 0.24) | 0.06 (0.03, 0.10) |
| ADD | | | | -0.13 (-0.29, 0.03) | 0.00 (-0.19, 0.19) | 0.29 (-0.25, 0.84) | 0.26 (0.13, 0.39) | -0.23 (-0.46, -0.00) | -0.03 (-0.23, 0.17) | 0.38 (0.02, 0.74) | 0.24 (-0.20, 0.68) | 0.02 (-0.76, 0.81) | 0.03 (-0.18, 0.23) | -0.00 (-0.51, 0.50) | 0.12 (-0.02, 0.27) | 0.04 (-0.13, 0.21) | 0.14 (-0.14, 0.42) | 0.21 (-0.04, 0.47) | NA | NA | 0.34 (0.13, 0.56) | 0.07 (-0.06, 0.20) |
| PSO | | | | | 0.84 (0.82, 0.87) | -0.01 (-0.15, 0.13) | 0.12 (0.08, 0.16) | 0.09 (0.05, 0.13) | 0.15 (0.11, 0.19) | 0.19 (0.10, 0.29) | 0.19 (0.10, 0.29) | 0.10 (-0.04, 0.24) | 0.02 (-0.03, 0.07) | 0.06 (-0.03, 0.15) | 0.13 (0.09, 0.16) | 0.17 (0.13, 0.21) | 0.09 (0.03, 0.16) | 0.14 (0.08, 0.20) | 0.10 (-0.05, 0.25) | 0.12 (-0.00, 0.25) | 0.15 (0.08, 0.22) | 0.02 (-0.01, 0.05) |
| PSA | | | | | | 0.03 (-0.15, 0.22) | 0.09 (0.04, 0.15) | 0.10 (0.04, 0.15) | 0.10 (0.04, 0.16) | 0.20 (0.08, 0.32) | 0.17 (0.04, 0.29) | 0.12 (-0.07, 0.31) | 0.05 (-0.01, 0.12) | 0.06 (-0.06, 0.18) | 0.33 (0.29, 0.38) | 0.27 (0.23, 0.32) | 0.21 (0.13, 0.30) | 0.21 (0.13, 0.29) | 0.01 (-0.21, 0.22) | 0.08 (-0.10, 0.25) | 0.08 (-0.01, 0.18) | 0.06 (0.02, 0.10) |
| DH | | | | | | | 0.58 (0.45, 0.72) | 0.21 (0.06, 0.36) | 0.02 (-0.18, 0.23) | -0.17 (-0.60, 0.27) | 0.39 (0.02, 0.76) | 0.00 (-0.55, 0.56) | -0.07 (-0.30, 0.16) | 0.25 (-0.07, 0.57) | 0.17 (0.03, 0.32) | -0.08 (-0.28, 0.12) | 0.22 (-0.02, 0.46) | 0.33 (0.12, 0.53) | 0.46 (-0.18, 1.10) | 0.04 (-0.72, 0.80) | 0.41 (0.16, 0.66) | 0.13 (-0.01, 0.26) |
| CED | | | | | | | | 0.13 (0.08, 0.18) | 0.10 (0.04, 0.15) | 0.17 (0.05, 0.29) | 0.10 (-0.02, 0.23) | 0.03 (-0.15, 0.22) | 0.02 (-0.04, 0.09) | -0.01 (-0.14, 0.11) | 0.07 (0.02, 0.12) | -0.02 (-0.08, 0.04) | 0.20 (0.12, 0.28) | 0.14 (0.07, 0.22) | 0.16 (-0.02, 0.35) | 0.09 (-0.08, 0.26) | 0.20 (0.11, 0.29) | 0.04 (0.00, 0.08) |
| UC | | | | | | | | | 0.57 (0.53, 0.61) | 0.20 (0.08, 0.31) | 0.18 (0.07, 0.30) | 0.49 (0.31, 0.68) | 0.01 (-0.05, 0.07) | 0.09 (-0.02, 0.21) | 0.08 (0.04, 0.13) | 0.22 (0.18, 0.27) | 0.01 (-0.08, 0.10) | 0.15 (0.07, 0.22) | 0.03 (-0.17, 0.23) | 0.14 (-0.02, 0.30) | 0.14 (0.05, 0.22) | -0.13 (-0.17, -0.09) |
| CRD | | | | | | | | | | 0.17 (0.04, 0.29) | -0.10 (-0.25, 0.06) | 0.39 (0.20, 0.58) | 0.06 (-0.01, 0.13) | 0.03 (-0.10, 0.15) | 0.07 (0.02, 0.13) | 0.27 (0.22, 0.32) | 0.04 (-0.06, 0.13) | 0.10 (0.02, 0.19) | 0.07 (-0.14, 0.28) | 0.11 (-0.06, 0.29) | 0.09 (-0.00, 0.19) | -0.05 (-0.09, -0.00) |
| AIH | | | | | | | | | | | 0.68 (0.48, 0.87) | 0.42 (0.07, 0.77) | 0.02 (-0.12, 0.16) | 0.19 (-0.05, 0.42) | 0.15 (0.04, 0.25) | 0.10 (-0.02, 0.22) | 0.39 (0.20, 0.58) | 0.20 (0.03, 0.36) | 0.27 (-0.13, 0.66) | 0.07 (-0.30, 0.44) | 0.06 (-0.15, 0.26) | 0.03 (-0.06, 0.13) |
| PBC | | | | | | | | | | | | 0.41 (0.09, 0.74) | 0.25 (0.11, 0.39) | -0.39 (-0.79, 0.01) | 0.06 (-0.05, 0.17) | -0.05 (-0.20, 0.09) | 0.26 (0.09, 0.43) | 0.22 (0.07, 0.37) | 0.08 (-0.32, 0.48) | NA | 0.18 (-0.02, 0.39) | 0.01 (-0.09, 0.12) |
| PSC | | | | | | | | | | | | | 0.09 (-0.12, 0.30) | 0.02 (-0.35, 0.39) | 0.14 (-0.02, 0.29) | 0.06 (-0.12, 0.24) | 0.07 (-0.22, 0.37) | 0.15 (-0.09, 0.39) | -0.04 (-0.70, 0.62) | -0.10 (-0.65, 0.46) | 0.23 (-0.07, 0.52) | 0.01 (-0.13, 0.15) |
| MS | | | | | | | | | | | | | | -0.04 (-0.19, 0.11) | 0.02 (-0.04, 0.08) | 0.08 (0.01, 0.14) | 0.08 (-0.02, 0.19) | 0.13 (0.04, 0.22) | 0.01 (-0.23, 0.25) | -0.05 (-0.26, 0.17) | 0.18 (0.07, 0.28) | 0.05 (0.00, 0.10) |
| MG | | | | | | | | | | | | | | | 0.21 (0.11, 0.30) | 0.09 (-0.03, 0.21) | 0.10 (-0.08, 0.28) | 0.24 (0.09, 0.40) | 0.39 (0.02, 0.76) | -0.29 (-0.73, 0.16) | 0.04 (-0.16, 0.24) | 0.09 (0.00, 0.18) |
| RA | | | | | | | | | | | | | | | | 0.26 (0.22, 0.30) | 0.39 (0.32, 0.45) | 0.43 (0.37, 0.49) | 0.22 (0.05, 0.38) | 0.39 (0.25, 0.54) | 0.16 (0.08, 0.24) | 0.06 (0.02, 0.09) |
| AS | | | | | | | | | | | | | | | | | 0.13 (0.04, 0.21) | 0.11 (0.03, 0.18) | -0.01 (-0.22, 0.20) | -0.24 (-0.48, 0.01) | 0.08 (-0.01, 0.17) | 0.01 (-0.04, 0.05) |
| SLE | | | | | | | | | | | | | | | | | | 0.61 (0.52, 0.70) | 0.47 (0.21, 0.72) | 0.24 (0.00, 0.47) | 0.09 (-0.06, 0.24) | 0.09 (0.02, 0.16) |
| SJS | | | | | | | | | | | | | | | | | | | 0.38 (0.13, 0.63) | 0.16 (-0.10, 0.43) | 0.11 (-0.03, 0.25) | 0.02 (-0.04, 0.09) |
| IIM | | | | | | | | | | | | | | | | | | | | 0.07 (-0.51, 0.65) | 0.31 (-0.04, 0.66) | 0.19 (0.03, 0.35) |
| GPA | | | | | | | | | | | | | | | | | | | | | 0.07 (-0.24, 0.38) | -0.00 (-0.14, 0.14) |
| SAR | | | | | | | | | | | | | | | | | | | | | | 0.09 (0.02, 0.16) |

**Supplemental Table S7. Disease combinations with adjusted genetic correlation close to zero.**

For the listed disease pairs, the estimated genetic correlations, adjusted for age and sex, did not differ significantly from zero, with 95% confidence intervals bounded within the range [–0.1, 0.1].

| **Disease 1** | **Disease 2** | **Genetic correlation** | **95% CI** | **P value** |
|---|---|---|---|---|
| T1D | AS | 0.01 | -0.04, 0.06 | 0.70 |
| GD | AS | -0.02 | -0.09, 0.04 | 0.44 |
| CED | AS | -0.02 | -0.08, 0.04 | 0.56 |
| HT | CRD | -0.02 | -0.05, 0.01 | 0.12 |
| PSO | MS | 0.02 | -0.03, 0.07 | 0.53 |
| GD | MS | 0.01 | -0.02, 0.05 | 0.82 |
| CED | MS | 0.02 | -0.04, 0.09 | 0.51 |
| UC | MS | 0.01 | -0.05, 0.07 | 0.75 |
| T1D | PSO | 0.03 | -0.01, 0.06 | 0.13 |
| UC | SLE | 0.01 | -0.08, 0.10 | 0.75 |

**Supplemental table S8. Overview of HLA risk alleles associated with selected autoimmune diseases**

Overview of commonly reported HLA associations for autoimmune diseases in populations of European ancestry. Diseases are arbitrarily ordered based on shared risk alleles (where applicable), although this ordering does not capture the full complexity of overlapping HLA associations. The strength and specificity of HLA associations vary across conditions; some diseases (e.g., celiac disease) are strongly linked to a limited set of HLA types, whereas others show associations with multiple alleles. Haplotypes are reported where alleles are in strong linkage disequilibrium and consistent with published findings.

| Disease | HLA risk alleles/haplotypes | PMID |
|---|---|---|
| GD | DRB1*03:01-DQA1*05:01-DQB1*02:01 | 19412418 |
| HT | DRB1*03:01-DQA1*05:01-DQB1*02:01, and HLA-DR4 | 19412418 |
| T1D | DRB1*03:01-DQA1*05:01-DQB1*02:01, and DRB1*04-DQA1*03:01-DQB1*03:02 | 19412418, 26212387 |
| ADD | DRB1*03:01-DQA1*05:01-DQB1*02:01, and DRB1*04-DQA1*03:01-DQB1*03:02 | 19412418, 33574239, 26212387 |
| DH | DRB1*03:01-DQA1*05:01-DQB1*02:01, and DRB1*04-DQA1*03:01-DQB1*03:02 | 31244841 |
| CED | DRB1*03:01-DQA1*05:01-DQB1*02:01, and DRB1*04-DQA1*03:01-DQB1*03:02 | 19412418, 26212387 |
| AIH | DRB1*03:01-DQA1*05:01-DQB1*02:01, and DRB1*04:01 | 26212387 |
| MG | DQB1*03:02, DQB1*02:01 | 19412418, 39537604 |
| PSC | A*01:01-C*07:01-B*08:01-DRB1*03:01-DQA1*05:01-DQB1*02:01, and DRB1*13:01-DQA1*01:03-DQB1*06:03 | 28695657 |
| RA | DRB1*04, *01, and *10 | 19412418, 31092910, 20061955 |
| PBC | DQB1*04:02-DQA1*04:01-DRB1*08:01 | 26212387 |
| CRO | DRB1*07:01-DQA1*02:01-DQB1, DRB1*04, and DRB1*01:03 | 26212387, 25559196 |
| ULC | DRB1*15:01, and DRB1*01:03 | 25559196, 26212387 |
| SLE | DRB1*03:02, and DRB1*15 | 19412418, 26212387 |
| SJS | DRB1*03:01, and DRB1*15:01 | 26212387 |
| MS | DRB1*15:01-DQA1*01:02-DQB1*06:02 | 26212387 |
| GPA | DPB1*04:01 | 38040271 |
| SAR | DRB1*11:01-DQA1*05:05-DQB1*03:01, DRB1:12:01-DQA1*05:05-DQB1*03:01, A*01:01–C*07:01–B*08:01–DRB1*03:01–DQA1*05:01–DQB1*02:01 | 25506722 |
| PSO | C*06:02-B*57-DRB1*07:01-DQA1*02:01 | 20953190, 34007822 |
| PSA | C*06:02-B*57-DRB1*07:01-DQA1*02:01 | 34007822 |
| IIM | DRB1*11,16,03 | 37769433 |
| AS | B*27 | 19412418 |

**Supplemental Table S9.** List of included autoimmune diseases with register-based criteria for inclusion.

| Disease | Abbreviation | Definition |
|---|---|---|
| Hashimoto's thyroiditis | HT | **Inclusion**<br>*Diagnoses*: ICD-8: 244.09, 245.03, 245.9. ICD-9: 244.8, 244.9, 245.2, 245.8, 245.9. ICD-10: E03.4-E03.9.<br>*Medications*: ≥2 dispensations of H03A<br>Either diagnoses or medication fulfilled.<br>**Exclusion**<br>*Diagnoses*: ICD-8: 193.99, 243.99, 244.00, 253.00–253.02, 253.09–253.11, 253.15, 253.18, 253.19, 253.90, 253.99. Or 242.00, 242.09 before fulfilled inclusion criteria. ICD-9: 242, 243, 244.0, 244.1, 244.2, 244.3, 253, 255.1. Or 241 before fulfilled inclusion criteria. ICD-10: E00.0-E00.9, E03.0-E03.3, E22.0-E22.9, E23.0-E23.7, E24.0, E89.0. Or E05.0-E05.9 before fulfilled inclusion criteria.<br>*Medications*: ≥1 dispensation of H03B, C01BD01, or N05AN before fulfilled inclusion criteria.<br>**Reference**: Thvilum, M., Brandt, F., Brix, T. H., & Hegedus, L. (2017). Month of birth is associated with the subsequent diagnosis of autoimmune hypothyroidism. A nationwide Danish register-based study. *Clin Endocrinol (Oxf)*, *87*(6), 832-837. https://doi.org/10.1111/cen.13425<br>*Difference*: We are missing C73 as exclusion criteria. |
| Graves' disease | GD | **Inclusion**<br>Diagnoses: ICD-8: 242.00, or 242.01, 242.08, 242.09. ICD-9: 242.0, 242.01. ICD-10: E05.0, H05.2, H06.2.<br>**Reference**: Brandt, F., Thvilum, M., Almind, D., Christensen, K., Green, A., Hegedus, L., & Brix, T. H. (2013). Graves' disease and toxic nodular goiter are both associated with increased mortality but differ with respect to the cause of death: a Danish population-based register study. *Thyroid*, *23*(4), 408-413. https://doi.org/10.1089/thy.2012.0500 |
| Type-1 diabetes | T1D | **Inclusion**<br>As indicated by being identified in the Swedish National Diabetes Register.<br>**Reference**: Eeg-Olofsson, K., Cederholm, J., Nilsson, P. M., Zethelius, B., Svensson, A. M., Gudbjornsdottir, S., & Eliasson, B. (2010). Glycemic control and cardiovascular disease in 7,454 patients with type 1 diabetes: an observational study from the Swedish National Diabetes Register (NDR). *Diabetes Care*, *33*(7), 1640-1646. https://doi.org/10.2337/dc10-0398 |
| Addison's disease | ADD | **Inclusion**<br>*Diagnoses*: ICD-8: 255.10. ICD-9: 255.4. ICD-10: E27.1, E27.2.<br>**Exclusions**<br>*Diagnoses*: ICD-8: 010-019, Y34.09, Y34.19, Y34.29, 036.11, 255.01, 255.02, 333.10, 758.10, 253. ICD-9: 010-018, 137, 036.3, 255.2, 255.5, 330.0, 759.1, 253. ICD-10: A15-A19, A39.1, B90, E23-E25, E27.3, E27.4, E35.1, E71.3, E89.3, E89.6, E220, E222, E228, E229, J65, P37.0, P54.4, Q89.1. |

| | | |
|---|---|---|
| | | **Reference**: Skov, J., Sundstrom, A., Ludvigsson, J. F., Kampe, O., & Bensing, S. (2019). Sex-Specific Risk of Cardiovascular Disease in Autoimmune Addison Disease-A Population-Based Cohort Study. *J Clin Endocrinol Metab*, *104*(6), 2031-2040. https://doi.org/10.1210/jc.2018-02298<br>*Difference*: We do not have B20-B24, B90, C74, C79, D35.0, D44.1, or J65 as exclusion. We do not require specific medications for inclusion. |
| Psoriasis | PSO | **Inclusion**<br>*Diagnoses*: ≥2 diagnoses (separate occasions) set at department of dermatology, rheumatology, or internal medicine; ICD-8: 696.09, 696.10, 696.19. ICD-9: 696.0, 696.1, ICD-10: L40.0-L40.2, L40.4, L5, L40.8, L40.9.<br>**Reference**: Lofvendahl, S., Theander, E., Svensson, A., Carlsson, K. S., Englund, M., & Petersson, I. F. (2014). Validity of diagnostic codes and prevalence of physician-diagnosed psoriasis and psoriatic arthritis in southern Sweden--a population-based register study. *PLoS One*, *9*(5), e98024. https://doi.org/10.1371/journal.pone.0098024<br>*Difference*: We do not have L40.5 as inclusion. |
| Psoriatic arthritis | PSA | **Inclusion**<br>*Diagnoses*: ≥2 diagnoses (separate occasions) set at department of dermatology, rheumatology, or internal medicine; ICD-8: 696.00. ICD-9: 696.0. ICD-10: L40.5, M07.0-M07.3, M09.0.<br>**Reference**: Lofvendahl, S., Theander, E., Svensson, A., Carlsson, K. S., Englund, M., & Petersson, I. F. (2014). Validity of diagnostic codes and prevalence of physician-diagnosed psoriasis and psoriatic arthritis in southern Sweden--a population-based register study. *PLoS One*, *9*(5), e98024. https://doi.org/10.1371/journal.pone.0098024 |
| Dermatitis herpetiformis | DH | **Inclusion**<br>*Diagnosis*: ICD-8: 693.99. ICD-9: 694.0. ICD-10: L13.0:<br>**Reference**: Askling et al. Gastroenterology. 2002 Nov;123(5):1428-35. doi: 10.1053/gast.2002.36585. PMID: 12404215. (Motsv. ICD-9) |
| Celiac disease | CED | **Inclusion**<br>*Diagnosis*: ICD-8: 269.00, 269.98. ICD-9: 579.0. ICD-10: K90.0.<br>**Reference**: Ludvigsson, J. F., Montgomery, S. M., & Ekbom, A. (2005). Celiac disease and risk of adverse fetal outcome: a population-based cohort study. *Gastroenterology*, *129*(2), 454-463. https://doi.org/10.1016/j.gastro.2005.05.065 |
| Ulcerative Colitis | UC | **Inclusion**<br>*Diagnoses*: ≥2 diagnoses (separate occasions); ICD-8: 563.10, 569.02. ICD-9: 556. ICD-10: K51.<br>**Reference**: Jakobsson, G. L., Sternegard, E., Olen, O., Myrelid, P., Ljung, R., Strid, H., Halfvarson, J., & Ludvigsson, J. F. (2017). Validating inflammatory bowel disease (IBD) in the Swedish National Patient Register and the Swedish Quality Register for IBD (SWIBREG). *Scand J Gastroenterol*, *52*(2), 216-221. https://doi.org/10.1080/00365521.2016.1246605<br>*Difference*: We allow simultaneous diagnosis of Ulcerative Colitis and Chron's disease. |
| Crohn's disease | CRD | **Inclusion**<br>*Diagnoses*: ≥2 diagnoses (separate occasions); ICD-8: 563.00. ICD-9: 555. ICD-10: K50. |

| | | |
|---|---|---|
| | | **Reference**: Jakobsson, G. L., Sternegard, E., Olen, O., Myrelid, P., Ljung, R., Strid, H., Halfvarson, J., & Ludvigsson, J. F. (2017). Validating inflammatory bowel disease (IBD) in the Swedish National Patient Register and the Swedish Quality Register for IBD (SWIBREG). *Scand J Gastroenterol*, *52*(2), 216-221. https://doi.org/10.1080/00365521.2016.1246605<br>*Difference*: We allow simultaneous diagnosis of Ulcerative Colitis and Chron's disease. |
| Autoimmune Hepatitis | AIH | **Inclusion**<br>*Diagnoses*: ICD-8: 573.0X 571.9. ICD-9: 571.4, 573.3. ICD-10: K75.4.<br>**Reference**: Stokkeland, K., Ludvigsson, J. F., Hultcrantz, R., Ekbom, A., Hoijer, J., Bottai, M., & Stephansson, O. (2016). Increased risk of preterm birth in women with autoimmune hepatitis - a nationwide cohort study. *Liver Int*, *36*(1), 76-83. https://doi.org/10.1111/liv.12901 |
| Primary Biliary Cholangitis | PBC | **Inclusion**<br>*Diagnoses*: ≥2 diagnoses (separate occasions); ICD-8: 571.90. ICD-9: 571.6.ICD-10: K74.3.<br>**Reference**: Myers, R. P., Shaheen, A. A., Fong, A., Wan, A. F., Swain, M. G., Hilsden, R. J., Sutherland, L., & Quan, H. (2010). Validation of coding algorithms for the identification of patients with primary biliary cirrhosis using administrative data. *Can J Gastroenterol*, *24*(3), 175-182. https://doi.org/10.1155/2010/237860 |
| Primary Sclerosing Cholangitis | PSC | **Inclusion**<br>*Diagnoses*: ICD-8: 575.05. ICD-9: 576.1. ICD-10: K83.0.<br>**Reference**: Bergquist, A., Montgomery, S. M., Bahmanyar, S., Olsson, R., Danielsson, A., Lindgren, S., Prytz, H., Hultcrantz, R., Loof, L. A., Sandberg-Gertzen, H., Almer, S., Askling, J., Ehlin, A., & Ekbom, A. (2008). Increased risk of primary sclerosing cholangitis and ulcerative colitis in first-degree relatives of patients with primary sclerosing cholangitis. *Clin Gastroenterol Hepatol*, *6*(8), 939-943. https://doi.org/10.1016/j.cgh.2008.03.016 |
| Multiple Sclerosis | MS | **Inclusion**<br>*Diagnoses*: ICD-8: 340.99. ICD-9: 340. ICD-10: G35.9.<br>**Reference**: Mason, K., Thygesen, L. C., Stenager, E., Bronnum-Hansen, H., & Koch-Henriksen, N. (2012). Evaluating the use and limitations of the Danish National Patient Register in register-based research using an example of multiple sclerosis. *Acta Neurol Scand*, *125*(3), 213-217. https://doi.org/10.1111/j.1600-0404.2011.01558.x |
| Myastenia Gravis | MG | **Inclusion**<br>*Diagnoses*: ICD-8: 733.9. ICD-9: 358.0, 358.1. ICD-10: G70.0.<br>*Medications*: ≥2 dispensations of N07AA02 or N07AA30.<br>Either diagnoses or medication fulfilled.<br>**Reference**: Pedersen, E. G., Hallas, J., Hansen, K., Jensen, P. E., & Gaist, D. (2011). Identifying patients with myasthenia for epidemiological research by linkage of automated registers. *Neuroepidemiology*, *37*(2), 120-128. https://doi.org/10.1159/000331481<br>And |

| | | |
|---|---|---|
| | | Fang, F., Sveinsson, O., Thormar, G., Granqvist, M., Askling, J., Lundberg, I. E., Ye, W., Hammarstrom, L., Pirskanen, R., & Piehl, F. (2015). The autoimmune spectrum of myasthenia gravis: a Swedish population-based study. *J Intern Med*, *277*(5), 594-604. https://doi.org/10.1111/joim.12310 |
| Rheumatoid Arthritis | RA | **Inclusion**<br>*Diagnoses*: ≥1 diagnosis if set at department of rheumatology or internal medicine, ≥2 diagnoses (separate occasions) if set by other specialist, for; ICD-8: 712.10, 712.20, 712.38, 712.39. ICD-9: 714.0-714.2, 719.3. ICD-10: M05, M06, M12.3.<br>**Reference**: Neovius, M., Simard, J. F., Askling, J., & group, A. s. (2011). Nationwide prevalence of rheumatoid arthritis and penetration of disease-modifying drugs in Sweden. *Ann Rheum Dis*, *70*(4), 624-629. https://doi.org/10.1136/ard.2010.133371 |
| Ankylosing Spondylitis | AS | **Inclusion**<br>*Diagnoses*: ICD-8: 712.40. ICD-9: 720.0. ICD-10: M45.<br>**Reference**: Askling, J., Klareskog, L., Blomqvist, P., Fored, M., & Feltelius, N. (2006). Risk for malignant lymphoma in ankylosing spondylitis: a nationwide Swedish case-control study. *Ann Rheum Dis*, *65*(9), 1184-1187. https://doi.org/10.1136/ard.2005.047514<br>*Difference*: We allow simultaneous diagnosis of Ankylosing Spondylitis and Rheumatoid Arthritis. |
| Systemic Lupus Erythematosus | SLE | **Inclusion**<br>*Diagnoses*: ≥2 diagnoses (separate occasions) set at department of rheumatology, internal medicine, nephrology, or dermatology; ICD-8: 734.1. ICD-9: 710.0. ICD-10: M32.<br>**Reference**: Simard, J. F., Sjowall, C., Ronnblom, L., Jonsen, A., & Svenungsson, E. (2014). Systemic lupus erythematosus prevalence in Sweden in 2010: what do national registers say? *Arthritis Care Res (Hoboken)*, *66*(11), 1710-1717. https://doi.org/10.1002/acr.22355<br>*Difference*: We did not require specific medications. |
| Sjögren's syndrome | SJS | **Inclusion**<br>*Diagnoses*: ICD-8: 734.90. ICD-9: 710.2. ICD-10: M35.0.<br>**Reference**: Vasaitis, L., Nordmark, G., Theander, E., Backlin, C., Smedby, K. E., Askling, J., Ronnblom, L., Sundstrom, C., & Baecklund, E. (2020). Population-based study of patients with primary Sjogren's syndrome and lymphoma: lymphoma subtypes, clinical characteristics, and gender differences. *Scand J Rheumatol*, *49*(3), 225-232. https://doi.org/10.1080/03009742.2019.1696403 |
| Idiopathic Inflammatory Myopathy | IIM | **Inclusion**<br>*Diagnoses*: ≥2 diagnoses (separate occasions) set at department of rheumatology, internal medicine, neurology, or dermatology; ICD-8: 716.10. ICD-9: 710.3, 710.4. ICD-10: M33.0, M33.1, M33.2, G72.4.<br>**Reference**: Meyer, A., Meyer, N., Schaeffer, M., Gottenberg, J. E., Geny, B., & Sibilia, J. (2015). Incidence and prevalence of inflammatory myopathies: a systematic review. *Rheumatology (Oxford)*, *54*(1), 50-63. https://doi.org/10.1093/rheumatology/keu289<br>And |

| | | |
|---|---|---|
| | | Svensson, J., Arkema, E. V., Lundberg, I. E., & Holmqvist, M. (2017). Incidence and prevalence of idiopathic inflammatory myopathies in Sweden: a nationwide population-based study. *Rheumatology (Oxford)*, *56*(5), 802-810. https://doi.org/10.1093/rheumatology/kew503 |
| Granulomatosis with polyangiitis | GPA | **Inclusion**<br>*Diagnoses*: ICD-8: 446.20. ICD-9: 446.4. ICD-10: M31.3.<br>**Reference**: Knight, A., Askling, J., & Ekbom, A. (2002). Cancer incidence in a population-based cohort of patients with Wegener's granulomatosis. *Int J Cancer*, *100*(1), 82-85. https://doi.org/10.1002/ijc.10444<br>*Difference*: We include outpatient specialists for ICD-10 (not available for reference). |
| Sarcoidosis | SAR | **Inclusion**<br>*Diagnoses*: ≥2 diagnoses (separate occasions); ICD-8: 135. ICD-9: 135. ICD-10: D86.<br>**Reference**: Ceder, S., Rossides, M., Kullberg, S., Eklund, A., Grunewald, J., & Arkema, E. V. (2021). Positive Predictive Value of Sarcoidosis Identified in an Administrative Healthcare Registry: A Validation Study. *Epidemiology*, *32*(3), 444-447. https://doi.org/10.1097/EDE.0000000000001323 |
| Appendicitis | APP | **Inclusion**<br>*Diagnoses*: ICD-8: 540, 541, 542. ICD-9: 540, 541, 542. ICD-10: K35, K36, K37. |

Note: For statistical reasons, no disease couples were considered mutually exclusive in the current definition.

**Supplemental Figure S1. Network analyses of adjusted genetic correlations across autoimmune diseases**. Adjusted for age and sex.Facets display networks generated using increasing thresholds for genetic correlations.

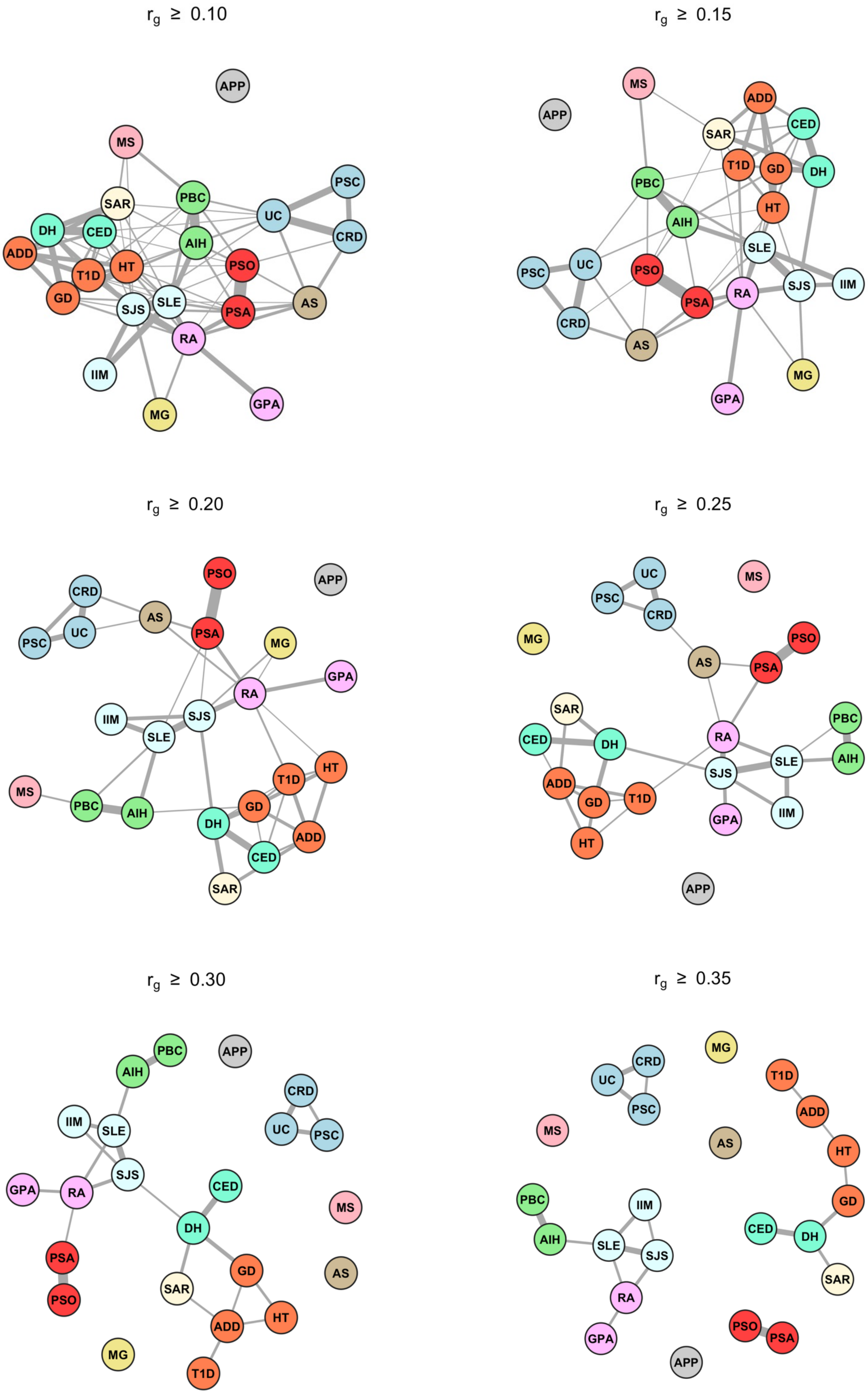

**Supplemental Figure S2. Circos plots of genetic correlations.** Based solely on significant genetic correlations. Facets display increasing thresholds for included genetic correlations.

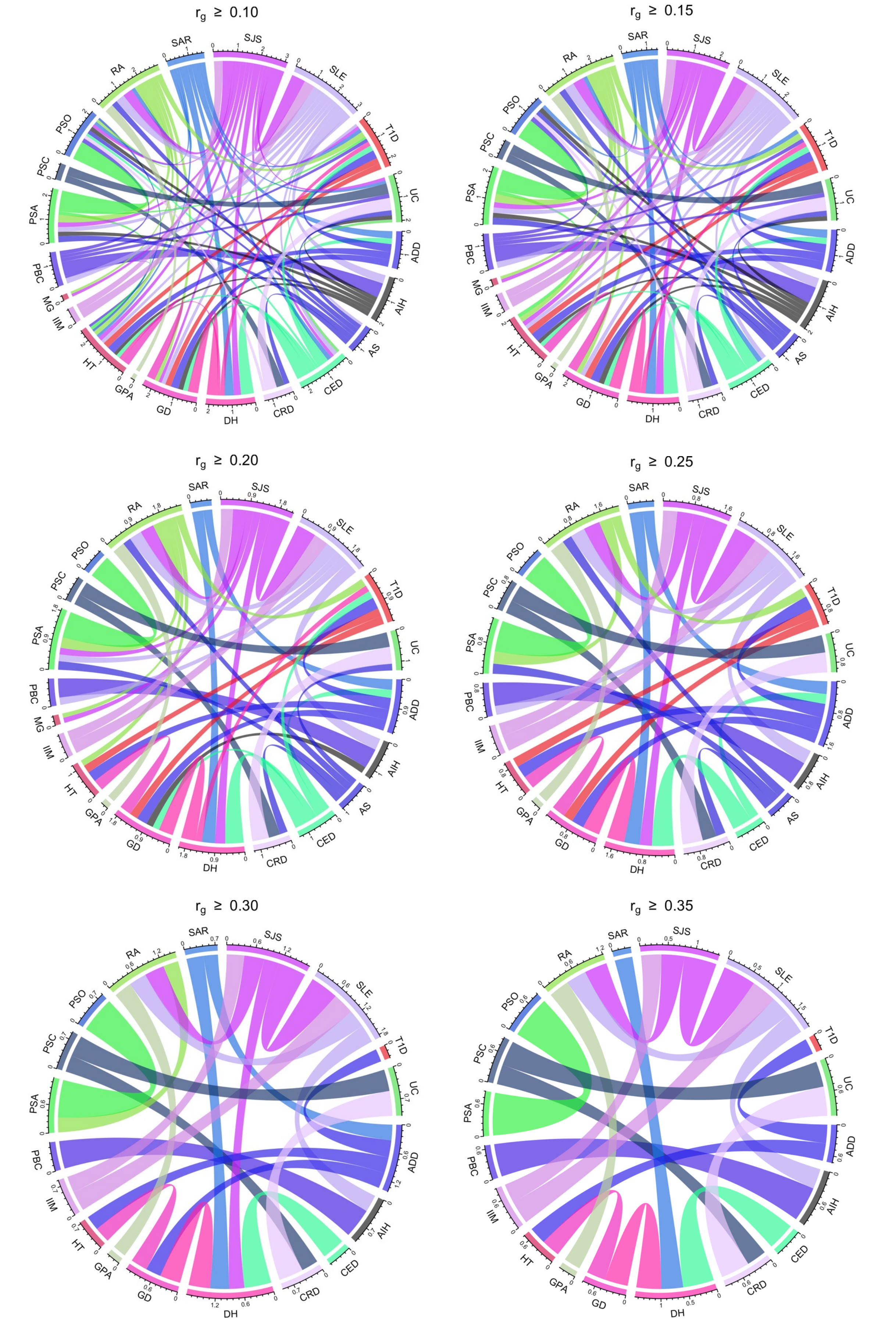

**Supplemental Figure S3. Genetic correlations vs GWAS based estimates**

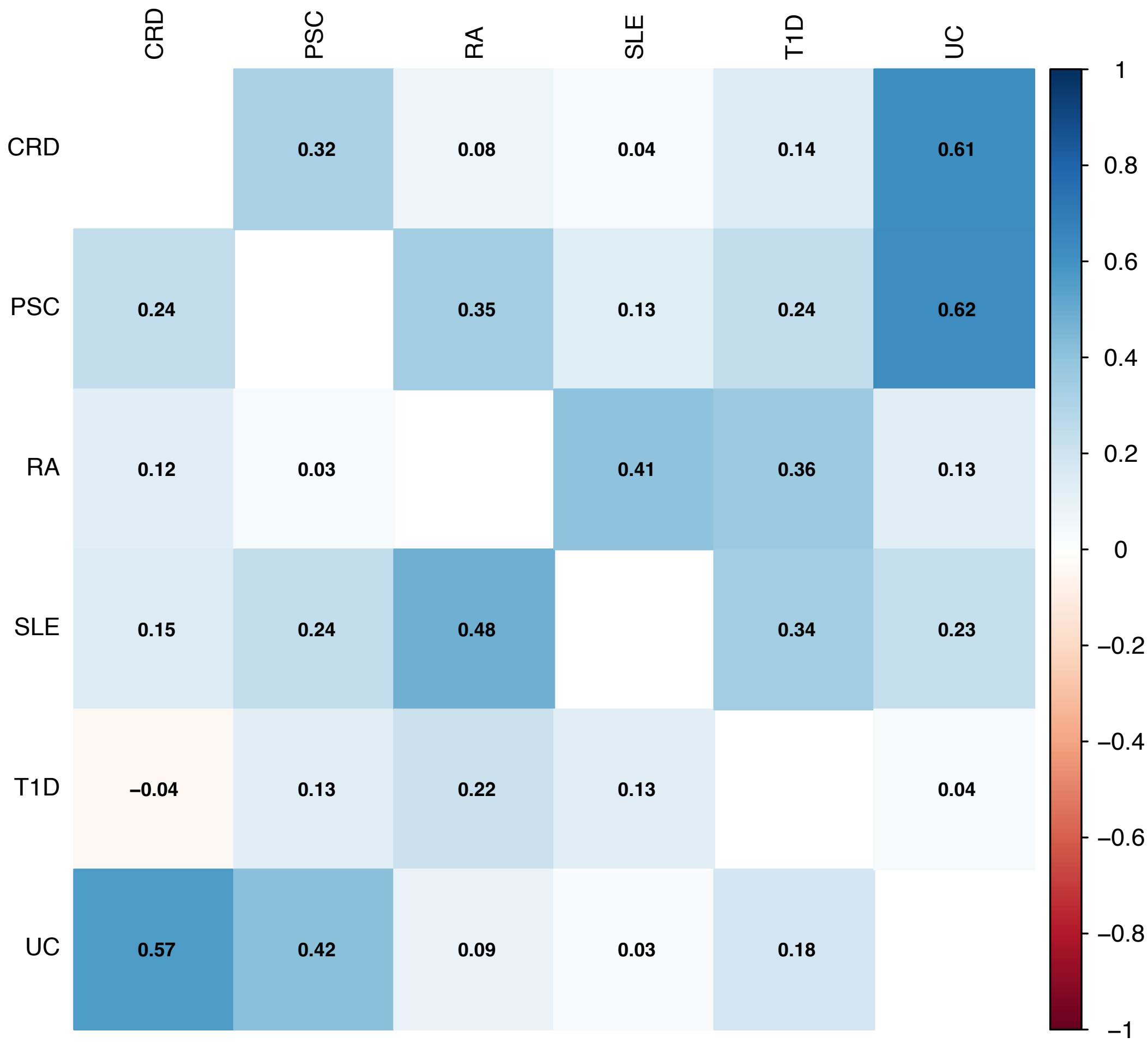


The matrix presents genetic correlations (rg) identified in our study (lower left triangle) alongside GWAS-based estimates from Demela et al. (PMID 37173304), derived using LDSC and Genomic SEM including the HLA region.

**Supplemental Figure S4. Correlation between epidemiological genetic correlations and GWAS based estimates**

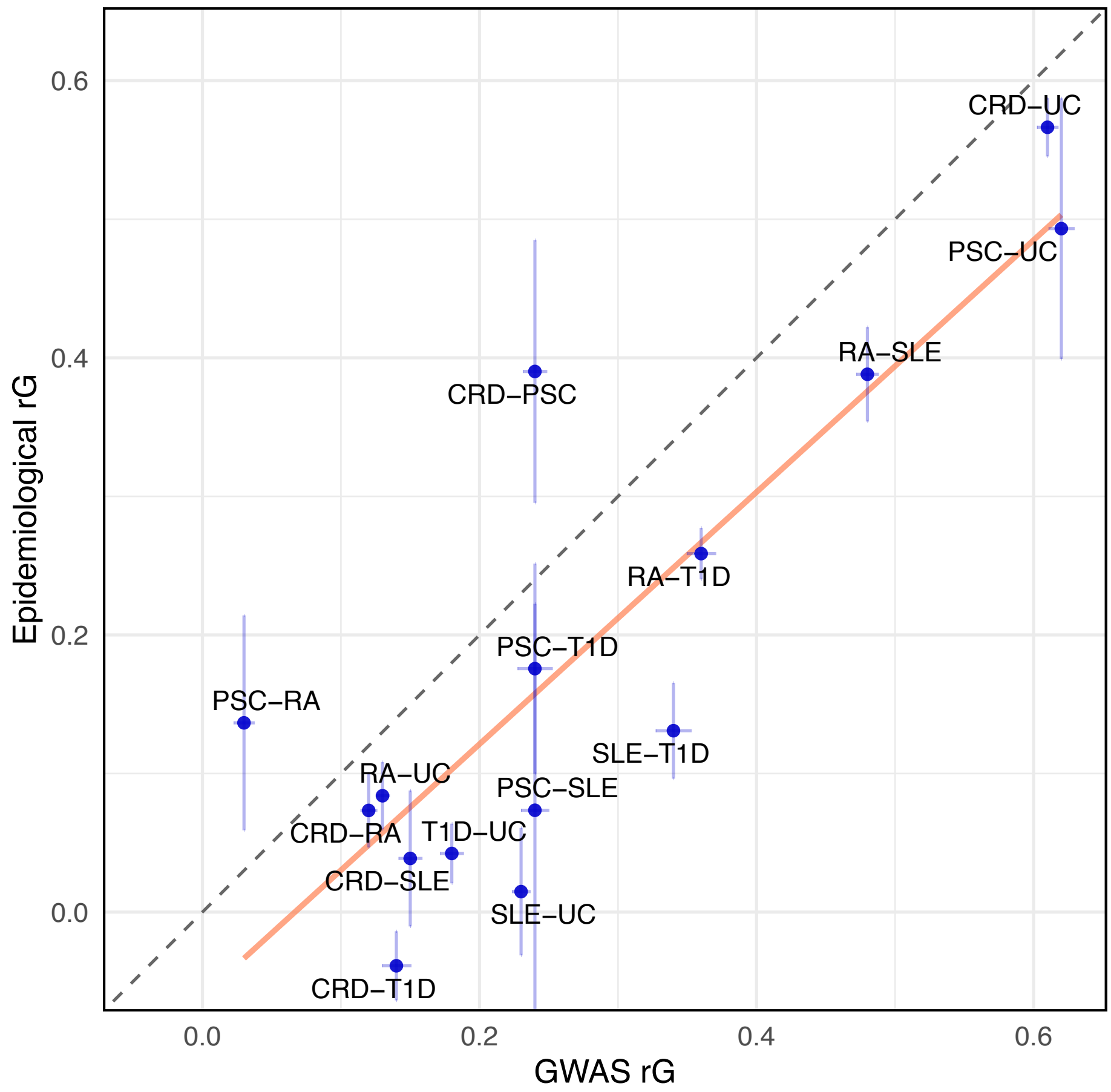


The scatterplot compares genetic correlations with GWAS-based estimates from Demela et al. (PMID 37173304), derived using LDSC and Genomic SEM including the HLA region. A linear model representing the data is depicted by a red line r = 0.84 (95% CI 0.57–0.95), while the equality line is delineated as a dashed grey line. Deming regression was used to account for measurement error in both variables. The error variance ratio was estimated from reported standard errors, and uncertainty was visualized with horizontal and vertical error bars.

**Supplemental Methods**

*Quantitative genetic model based on tetrachoric correlations*

We used a simplified method to estimate the genetic ($r_g$) and unique environmental ($r_e$) correlations. Because the dataset was too large for standard model fitting approaches, we instead relied on separately estimated tetrachoric correlations. And, even though we had a large dataset, because some of the diseases were so rare, we only used full siblings for estimation. Thus, we were only able to estimate one source of familiality of diseases, in addition to individually unique environment.

*Derivation of $r_g$ and its standard error based on correlations*

Assume that, for full sibs, the intraclass correlation (ICC), i.e., the correlation between relatives within one disease, is

$$ICC_i = \frac{1}{2} a_i^2$$

Where $i$ represents a disease. And, assume the cross-sibling cross-trait correlation (CSCT) is

$$CSCT_{ij} = \frac{1}{2} a_i r_g a_j$$

Where $i$ and $j$ represents different diseases in the different siblings. Then we can find an estimate of the square root of additive genetic contribution to cross-sibling correlation as

$$a_i = \sqrt{2 \cdot ICC_i}$$

And, we can find genetic correlation as

$$r_g = f\left(ICC_{\mathrm{i}}, ICC_j, CSCT_{ij}\right) = \frac{2 \cdot CSCT_{ij}}{a_{\mathrm{i}} \cdot a_j} = \frac{2 \cdot CSCT_{ij}}{\sqrt{2 \cdot ICC_{\mathrm{i}}}\sqrt{2 \cdot ICC_{\mathrm{j}}}} = \frac{CSCT_{ij}}{\sqrt{ICC_{\mathrm{i}}}\sqrt{ICC_{\mathrm{j}}}}$$

For standard errors we can use the delta method. First, the jacobian is

$$J = \begin{bmatrix} \frac{\partial}{\partial ICC_{\mathrm{i}}} r_g \\ \frac{\partial}{\partial ICC_{\mathrm{j}}} r_g \\ \frac{\partial}{\partial CSCT_{ij}} r_g \end{bmatrix} = \begin{bmatrix} -\frac{1}{2} \frac{CSCT_{ij}}{ICC_{\mathrm{i}}^{3/2} \cdot \sqrt{ICC_{\mathrm{j}}}} \\ -\frac{1}{2} \frac{CSCT_{ij}}{\sqrt{ICC_{\mathrm{i}}} \cdot ICC_{\mathrm{j}}^{3/2}} \\ \frac{1}{\sqrt{ICC_{\mathrm{i}}} \cdot \sqrt{ICC_j}} \end{bmatrix}$$

And the standard error is thus

$$SE_{r_g} = \sqrt{J^T \Sigma J}$$

Where $\Sigma$ is observed Covariance matrix of $\left[ICC_{\mathrm{i}}, ICC_{\mathrm{j}}, CSCT\right]$.

We used the library ‘OpenMx’ in software R to derive the tetrachoric correlations and their covariance.
